# Genomic inversions and *GOLGA* core duplicons underlie disease instability at the 15q25 locus

**DOI:** 10.1371/journal.pgen.1008075

**Published:** 2019-03-27

**Authors:** Flavia A. M. Maggiolini, Stuart Cantsilieris, Pietro D’Addabbo, Michele Manganelli, Bradley P. Coe, Beth L. Dumont, Ashley D. Sanders, Andy Wing Chun Pang, Mitchell R. Vollger, Orazio Palumbo, Pietro Palumbo, Maria Accadia, Massimo Carella, Evan E. Eichler, Francesca Antonacci

**Affiliations:** 1 Dipartimento di Biologia, Università degli Studi di Bari “Aldo Moro”, Bari, Italy; 2 Department of Genome Sciences, University of Washington School of Medicine, Seattle, WA, United States of America; 3 The Jackson Laboratory, Bar Harbor, ME, United States of America; 4 European Molecular Biology Laboratory (EMBL), Genome Biology Unit, Meyerhofstraße 1, Heidelberg, Germany; 5 Bionano Genomics, San Diego, CA, United States of America; 6 Medical Genetics Unit, IRCCS Casa Sollievo della Sofferenza, San Giovanni Rotondo (FG), Italy; 7 Medical Genetics Service, Hospital “Cardinale G. Panico”, Via San Pio X n°4, Tricase, LE, Italy; 8 Howard Hughes Medical Institute, University of Washington, Seattle, WA, United States of America; Duke University, UNITED STATES

## Abstract

Human chromosome 15q25 is involved in several disease-associated structural rearrangements, including microdeletions and chromosomal markers with inverted duplications. Using comparative fluorescence *in situ* hybridization, strand-sequencing, single-molecule, real-time sequencing and Bionano optical mapping analyses, we investigated the organization of the 15q25 region in human and nonhuman primates. We found that two independent inversions occurred in this region after the fission event that gave rise to phylogenetic chromosomes XIV and XV in humans and great apes. One of these inversions is still polymorphic in the human population today and may confer differential susceptibility to 15q25 microdeletions and inverted duplications. The inversion breakpoints map within segmental duplications containing core duplicons of the *GOLGA* gene family and correspond to the site of an ancestral centromere, which became inactivated about 25 million years ago. The inactivation of this centromere likely released segmental duplications from recombination repression typical of centromeric regions. We hypothesize that this increased the frequency of ectopic recombination creating a hotspot of hominid inversions where dispersed *GOLGA* core elements now predispose this region to recurrent genomic rearrangements associated with disease.

## Author summary

Human chromosome 15 derived from a fission event that occurred in the ancestor of great apes. Following inactivation of the ancestral centromere at 15q25 a dispersal of segmental duplications took place, providing templates for ectopic recombination and predisposing the region to genomic instability. Different disease-associated microdeletions and chromosomal markers have been described with breakpoints mapping within these segmental duplications. To gain insight into the instability at 15q25, we sought to analyze this region in human and nonhuman primates using multiple genomics techniques and demonstrated the presence of two independent inversion events that occurred during great apes evolution. One of these inversions is still polymorphic in humans and may cause, in conjunction with a *GOLGA* core duplicon—a ~14 kbp chromosome 15 repeat, susceptibility to non-allelic homologous recombination leading to pathogenic recurrent rearrangements. Our results support the existence of a strong relationship between inversions and core duplicons and reinforce the hypothesis that *GOLGA* repeats play a fundamental role both in disease and evolution.

## Introduction

Human chromosome 15 was generated by the chromosome fission of an ancestral submetacentric chromosome in the ancestor of great apes. Macaque chromosome 7 represents the ancestral state, as more distantly related organisms have the same configuration [1]. The ancestral centromere at 15q25 inactivated and lost any centromeric satellites, whereas a large cluster of segmental duplications persisted [2]. Following inactivation of the ancestral centromere, the constraint against recombination in this area was likely weakened [3–5], providing an environment permissive to non-allelic rearrangements that promoted the dispersal of the segmental duplications.

The 15q25 locus approximately corresponds to the position of the ancestral centromere [2] and is an unstable region of the human genome enriched in segmental duplications containing the *GOLGA* core duplicon, a ~14 kbp chromosome 15 repeat [6]. Cores represent ancestral duplications where additional duplication blocks have been formed around, and correspond to the expansion of gene families, some of which show signatures of positive selection [7]. *GOLGA* belongs to the golgin subfamily of coiled-coil proteins associated with the Golgi apparatus. These genes appear to have roles in membrane traffic and Golgi structure, but their precise function is in most cases unclear. *GOLGA* encodes a primate-specific gene family that expanded over the last 20 million years [8, 9]. Human chromosome 15 contains nearly 40 copies of the *GOLGA* core element [6], dispersed to multiple locations across the long arm of chromosome 15. *GOLGA* is one of 14 core duplicons associated with the burst of interspersed segmental duplications in the human–great ape ancestral lineage [10, 11] and the most enriched sequence associated with segmental blocks promoting evolutionary rearrangements in primates [12–14] and disease instability, including Prader-Willi/Angelman syndromes, 15q13 microdeletions and 15q24 microdeletions [13, 15–18].

The 15q25 region represents a high-risk locus for pediatric neurologic disease with variable outcomes [2, 19–29]. Different microdeletions and chromosomal markers with inverted duplications of chromosome 15 all have breakpoints mapping within a 3.3 Mbp region containing three blocks of segmental duplications of 350 kbp, 560 kbp and 115 kbp in size. The middle block contains a gap in the last release of the human reference genome (GRCh38/hg38) suggesting the possible presence of different structural haplotypes in this locus. We characterized the organization of this region in human and nonhuman primate genomes by conducting a detailed analysis by fluorescence *in situ* hybridization (FISH), single-cell strand-sequencing (Strand-seq), high-quality finished sequencing using PacBio single-molecule, real-time (SMRT) sequencing technology, and optical mapping (Bionano) in order to understand the extent of human genetic variation, its origin, and impact on disease.

## Results

### Characterization of chromosome 15q25 inversion variants

**Fig 1:**
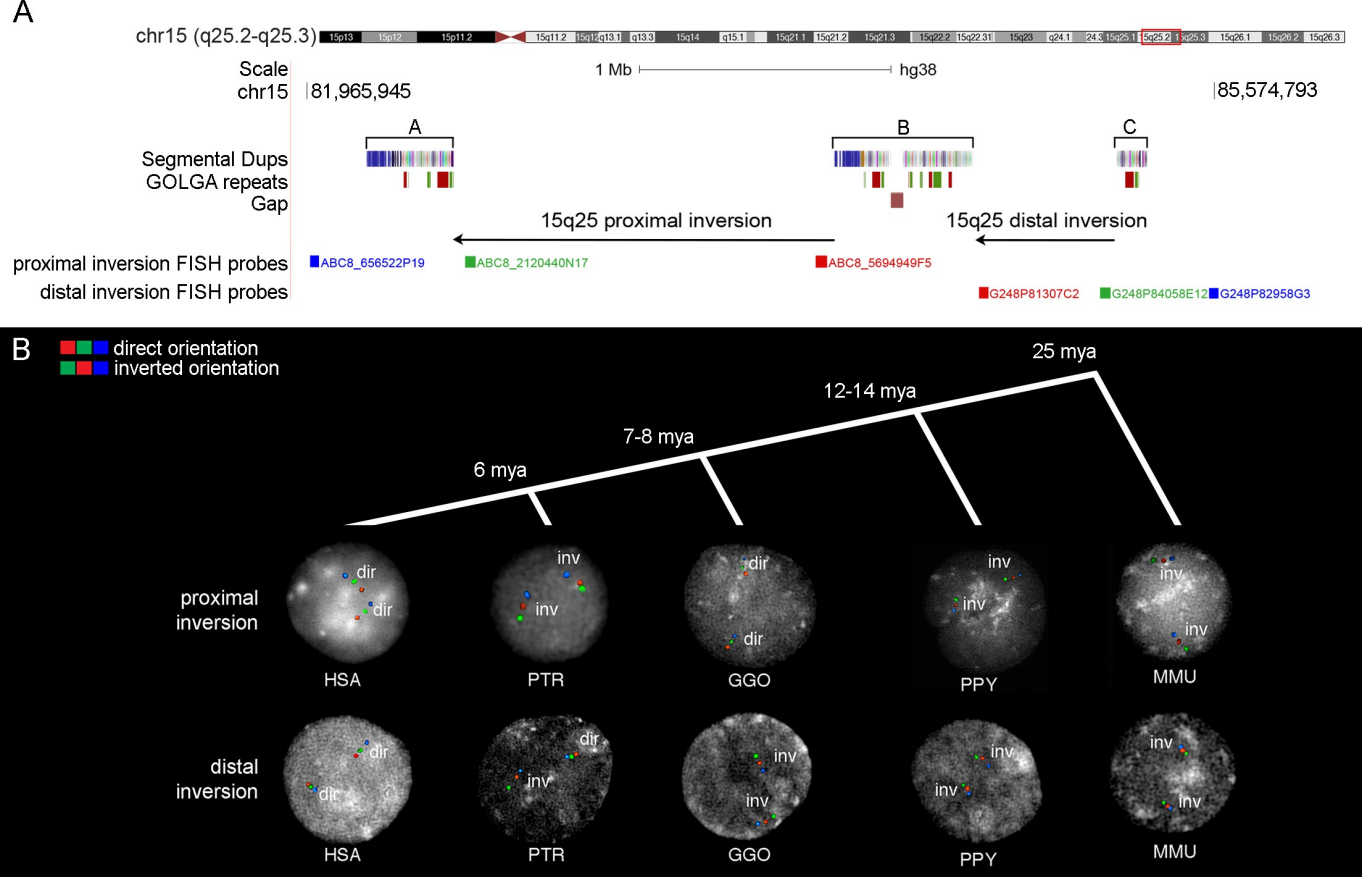
Experimental validation of genomic inversions at 15q25. (A) UCSC Genome Browser view of the 15q25 region. Segmental duplication blocks A, B and C are shown with colored boxes. *GOLGA* repeat locations from blat analyses of *GOLGA2P10* and *GOLGA6L5P* are depicted with green and red boxes mapping on the plus or minus strand, respectively. Proximal and distal tested inversions are shown with black arrows and fosmid clones used for FISH experiments on interphase nuclei are indicated with colored blocks followed by the fosmid names. Segmental duplication colors show the ancestral origins of duplications based on comparison with mammalian groups assigned by DupMasker [76]. (B) FISH results on interphase nuclei for proximal and distal inversions in each analyzed species. The color order indicates probes relative orientation, with red-green-blue signals showing haplotypes in direct orientation and green-red-blue signals showing inverted haplotypes. FISH analyses of the proximal inversion show that macaque, orangutan, and chimpanzee are all inverted when compared to the human reference genome orientation, while all gorillas are in direct orientation. The distal inversion is polymorphic within the chimpanzee population, while all the other species are inverted in the homozygous state when compared to human. Timing of species divergences is also shown at the top (mya = million years ago). HSA = *Homo sapiens*; PTR = *Pan troglodytes*; GGO = *Gorilla gorilla*; PPY = *Pongo pygmaeus*; MMU = *Macaca mulatta*.

Three duplication blocks, containing *GOLGA* repeats, map at the 15q25 region (S1 Table), with the middle one (block B) containing a gap in the reference genome (Fig 1A), suggesting that alternative structural configurations might exist within the human population. To gain insight into the instability associated to disease and evolutionary rearrangements at 15q25 we sought to characterize the organization of this region in more detail in human and nonhuman primate genomes. Using FISH experiments in interphase nuclei, we tested 22 HapMap individuals from different populations for the presence of two putative inversions: the proximal inversion of 1.5 Mbp between duplication blocks A and B (chr15:82534139–84045983) and the distal inversion of 600 kbp between duplication blocks B and C (chr15:84596420–85169772). All individuals tested for both distal and proximal inversions were in direct orientation (Fig 1B; S2 Table).

**Fig 2:**
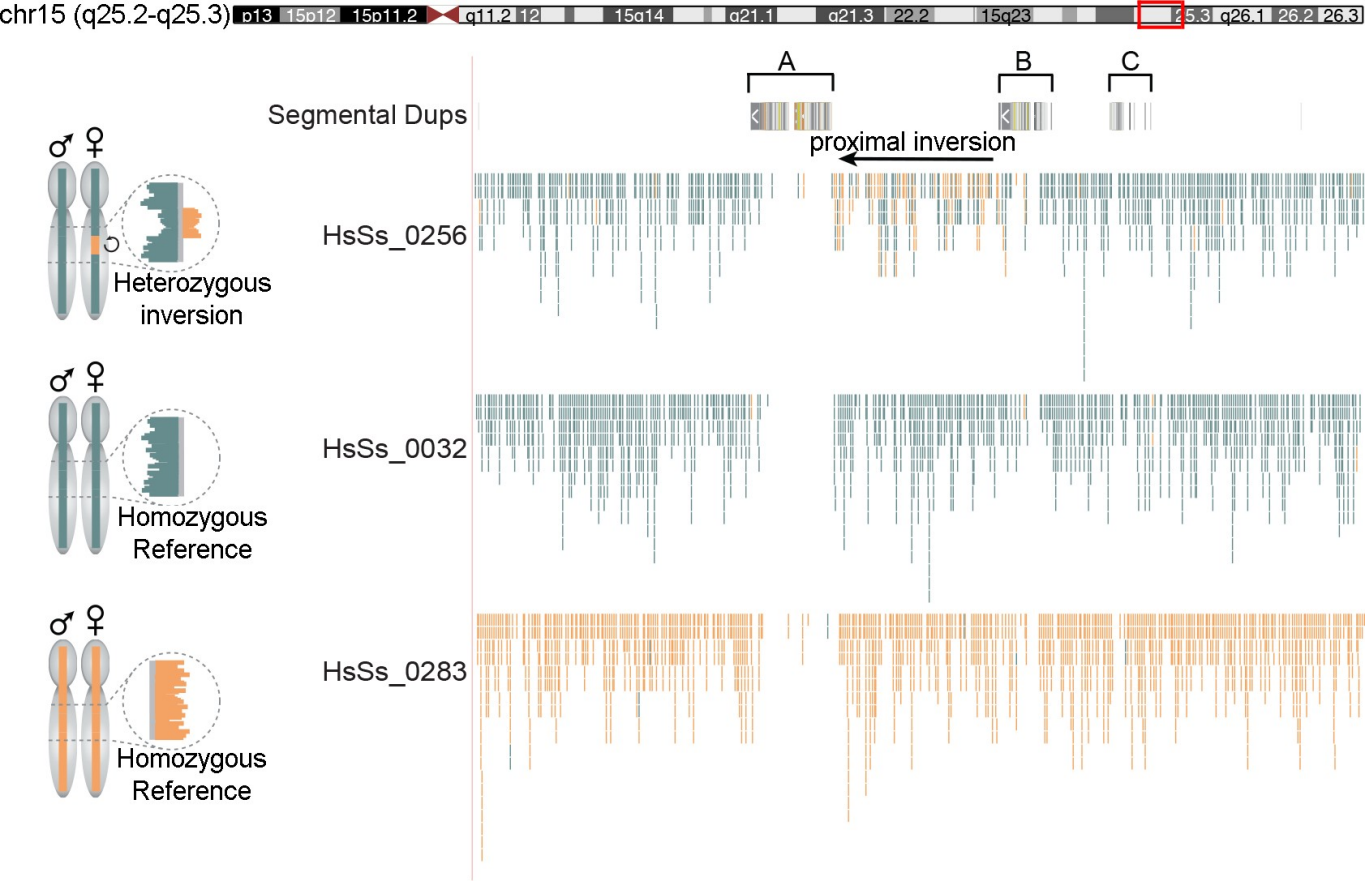
Strand-seq of the proximal inversion at 15q25. On the left, ideograms of expected Strand-seq results for each possible inversion genotype are shown. On the right, a UCSC Genome Browser view (coordinates lifted to GRCh37/hg19) of Strand-seq data, BED-formatted and uploaded as custom tracks, of the three libraries is shown. For each cell, aligned reads are indicated as individual lines in Crick (teal) or Watson (orange) state. In the library on the top (HsSs_0256) mixed Watson and Crick reads at the proximal inversion (black arrow) indicate the heterozygosity of the region while in the others a direct orientation of the region is shown.

In order to gain more information regarding the presence and frequency of the 15q25 inversions, we investigated published Strand-seq data from 47 libraries from a pool of 353 separate cord blood and bone marrow donors [30]. In Strand-seq libraries, inversions cause a segmental change in strand orientation at the inverted locus, which allows inversions to be directly visualized and genotyped in single-cell data [30]. One out of 22 informative libraries was heterozygous for the proximal inversion, while all the others were in direct orientation. All 22 libraries were in direct orientation for the distal region (Fig 2). In total, we tested 88 chromosomes (44 chromosomes by FISH analyses and 44 chromosomes by Strand-seq) and showed that only one out of the 88 chromosomes was in inverted orientation for the proximal region (inversion allele frequency of 1.14%) (Table 1).

**Table 1:** Summary of inversion frequencies in human and nonhuman primates. Inversion frequencies for the proximal and distal region based on FISH, Strand-seq and optical mapping analyses are shown. The number of individuals tested for each species is shown in parenthesis.

Species	15q25 proximal inversion frequency	15q25 distal inversion frequency
**Human (n = 44)**	1.14%	0%
**Chimpanzee (n = 9)**	100%	39%
**Gorilla (n = 5)**	0%	100%
**Orangutan (n = 5)**	100%	100%
**Macaque (n = 1)**	100%	100%

### Closing the gap in the human reference assembly

In order to close the gap in the reference genome we generated a map of contiguous clones from the CH17 BAC library from a hydatidiform mole-derived (haploid) human cell line (CHM1hTERT) [31]. Using BAC-end sequence pair mapping, we constructed a contiguous set of four BAC clones (S3 Table) and then performed SMRT sequencing. We generated a 657 kbp sequence contig spanning the B block of segmental duplications and closed the gap in this region. Miropeats analysis of the CH17 contig versus the human hg38 reference showed that the hydatidiform mole is in direct orientation for the proximal inversion and highlighted the presence of 64 kbp redundant sequence, containing *GOLGA* repeats, that was represented twice within the reference (S1 Fig).

### Evolutionary analyses

**Fig 3:**
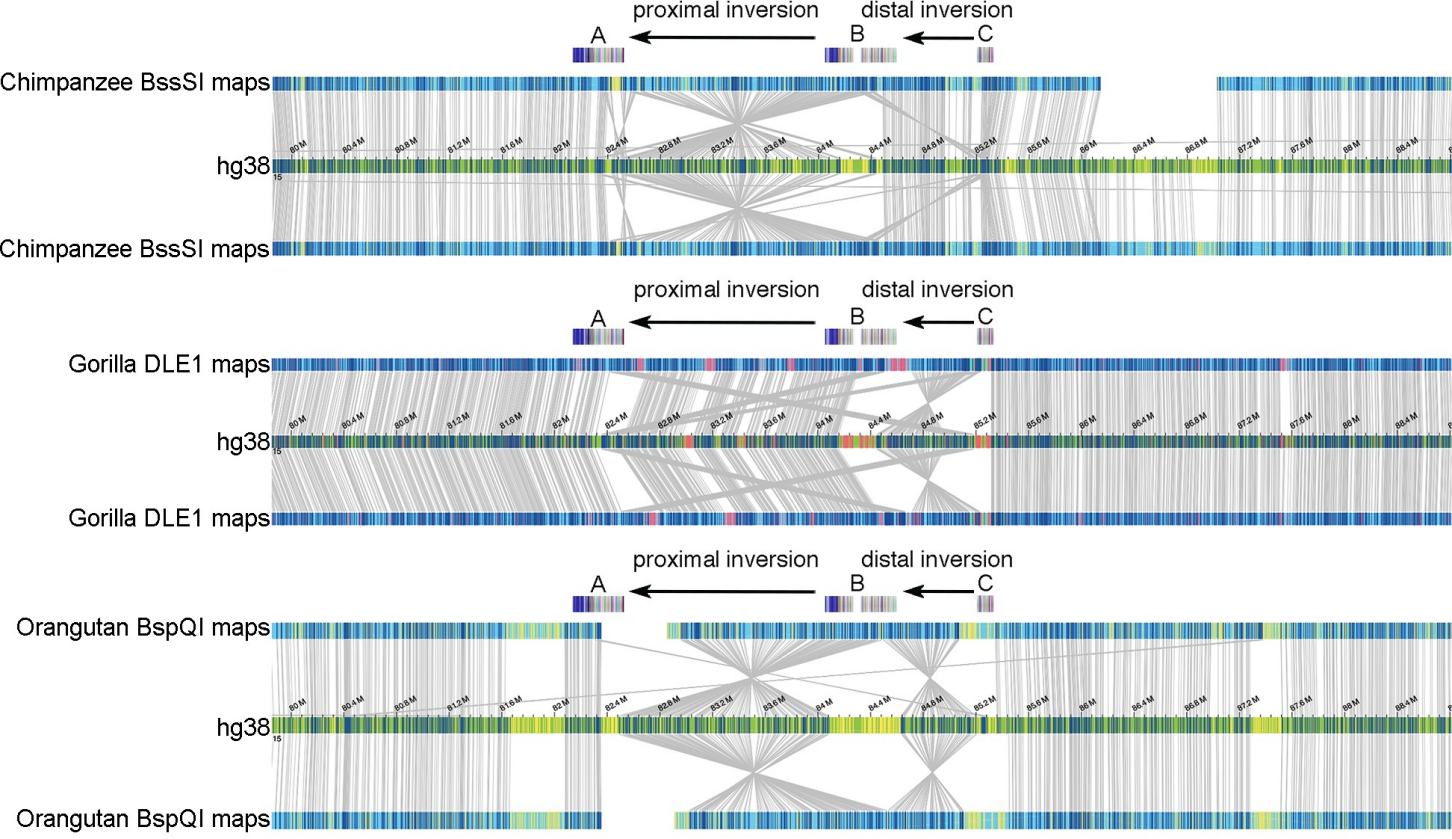
Bionano analysis of the 15q25 region. Bionano optical mapping data of three great ape genomes at the 15q25 locus. The two black arrows in each plot denote the two loci where inversions are observed between the apes and human. Segmental duplication blocks A, B and C are also shown with colored boxes. In each display, the top and bottom maps represent the two alleles of the *de novo* assembled genomes for each species with respect to the human reference assembly (hg38 track). The individual labels represent the positions of the label motifs of the enzyme used. The top panel shows the alignments of the assembled Nt.BssSI genome maps of a chimpanzee sample. The blue labels are the aligned labels, whereas the yellow ones are unaligned labels. The middle panel shows gorilla maps generated by DLE-1 enzyme, and the blue and red labels represent aligned and unaligned labels, respectively. Finally, the bottom panel illustrates how an orangutan genome—constructed using Nb.BspQI—is aligned to human, with blue representing aligned labels and yellow the unaligned ones.

In order to investigate the ancestral configuration of the 15q25 region, we compared the orientation of the proximal and distal regions in human with other nonhuman primate species. We tested for the presence of the proximal inversion between duplication blocks A and B by FISH analysis of cell lines from eight chimpanzees (*Pan troglodytes*), four gorillas (*Gorilla gorilla*), four orangutans (*Pongo pygmaeus*), and one macaque (*Macaca mulatta*) (Fig 1B; S2 Table). Moreover, we analyzed Bionano optical mapping data of DNA from one chimpanzee (*Pan troglodytes*), one gorilla (*Gorilla gorilla*) and one orangutan (*Pongo abelii*) (Fig 3; S2 Table). Chimpanzee, orangutan and macaque were found to be inverted when compared to the human reference genome orientation suggesting that this represents the likely ancestral state, while all gorillas were in direct orientation, similar to humans. We conclude that the proximal inversion likely occurred in the human–African great ape ancestor and the chimpanzee configuration may represent incomplete lineage sorting of the ancestral state or the inversion may have occurred at multiple times during great ape evolution as a result of recurrent mutation events involving the duplicated sequences.

Next, we tested for the presence of the distal inversion between duplication blocks B and C by FISH analysis of eight chimpanzees (*Pan troglodytes*), four gorillas (*Gorilla gorilla*), four orangutans (*Pongo pygmaeus*), and one macaque (*Macaca mulatta*). We found this inversion to be widely polymorphic within the chimpanzee population, while all other nonhuman species were inverted in the homozygous state (Fig 1B; S2 Table). Bionano optical mapping data of DNA from one gorilla (*Gorilla gorilla*) and one orangutan (*Pongo abelii*) show that these individuals are inverted in the homozygous state while chimpanzee (*Pan troglodytes*) is in direct orientation for both haplotypes (Fig 3; S2 Table). These data suggest that the inversion occurred in the human–chimpanzee ancestor and is still polymorphic in chimpanzee with a 39% allele frequency (Table 1).

### Duplication analysis

Given the central role of the duplications in both microdeletions and the evolution of inversions [13, 32–38], we compared the duplication architecture among primate species. Using BAC-end sequence pair mapping, we selected three clones from the CH276 orangutan BAC library and one clone from the CH251 chimpanzee library, which spanned the 600 kbp distal inversion breakpoint between blocks B and C, and then sequenced them using PacBio SMRT sequencing (S3 Table; S2 Fig). In orangutan we generated a ~400 kbp sequence contig and compared this with the human reference assembly. In addition, to confirm the presence of the inversion between duplication blocks B and C, we identified that the inversion would create an ancestral B/C block-hybrid. Sequence analysis demonstrates that this block is missing one copy of the *GOLGA* core duplicon identified in the human B block and two copies in the human C block (S2A Fig). We queried GenBank and identified two additional clones (250 kbp sequence contig) from CH250 macaque BAC library spanning this ancestral B/C block. Comparison of the B/C block-hybrid between orangutan and macaque shows that they both have two copies of *GOLGA* repeats in this region and, therefore, are missing a total of three copies with respect to the human orthologous regions (S2B Fig). The ancestral orientation of the duplication block is itself inverted in the macaque relative to orangutan (S2B Fig), suggesting a restructuring of the duplication architecture of the region during primate evolution. Finally, Miropeats analyses of a CH251 clone from a chimpanzee (Clint) in homozygous direct orientation for the distal inversion between B/C blocks shows that human and chimpanzee are collinear and both have two copies of *GOLGA* repeats for this region (S2C Fig).

### *GOLGA* copy number analysis in human and nonhuman primates

To further investigate the copy number of the *GOLGA* core duplicons in humans and primates, we performed a BLAT analysis using *GOLGA2P10* and *GOLGA6L5P* exon sequences. We generated a map of *GOLGA* repeats in the 15q25 human region, which allowed us to identify 24 repeats in the latest human reference genome assembly (S1 Table; S3 Fig). We performed the same analysis on primate reference genomes (Clint_PTRv2/panTro6, gorGor4.1/gorGor4, Susie_PABv2/ponAbe3 and BCM Mmul_8.0.1/rheMac8) and found 11 repeats in chimpanzee and gorilla and 13 in orangutan and macaque (S1 Table). However, the exact number of copies could not be determined due to the presence of gaps in the assembly region (3 gaps in chimpanzee, 67 in gorilla, 1 in orangutan and 20 in macaque). To determine the extent to which gaps affected the observed difference in copy number we performed a parallel analysis using four assemblies all built using the same PacBio sequencing technology and FALCON assembly method (chimpanzee Clint_PTRv2/panTro6, gorilla Susie3, orangutan Susie_PABv2/ponAbe3, and human PacBioCHM1_r2). Using whole genome alignments of these four assemblies to GRCh38 we found that of the 24 *GOLGA* repeats in the 15q25 region, 21 are found in the human *de novo* assembly (CHM1) which is twice the number of copies compared to other primate species (9 in chimpanzee, 10 in gorilla and 8 in orangutan) (S4 Table). The high recovery rate of *GOLGA* repeats in CHM1 as compared to GRCh38 (21/24) suggests that the large majority of these sequences are resolved in *de novo* PacBio assemblies and that the reduced copy number in Clint_PTRv2/panTro6, Susie3, and Susie_PABv2/ponAbe3 is not due to gaps in their respective assemblies.

### Pathogenic rearrangements at 15q25

**Fig 4:**
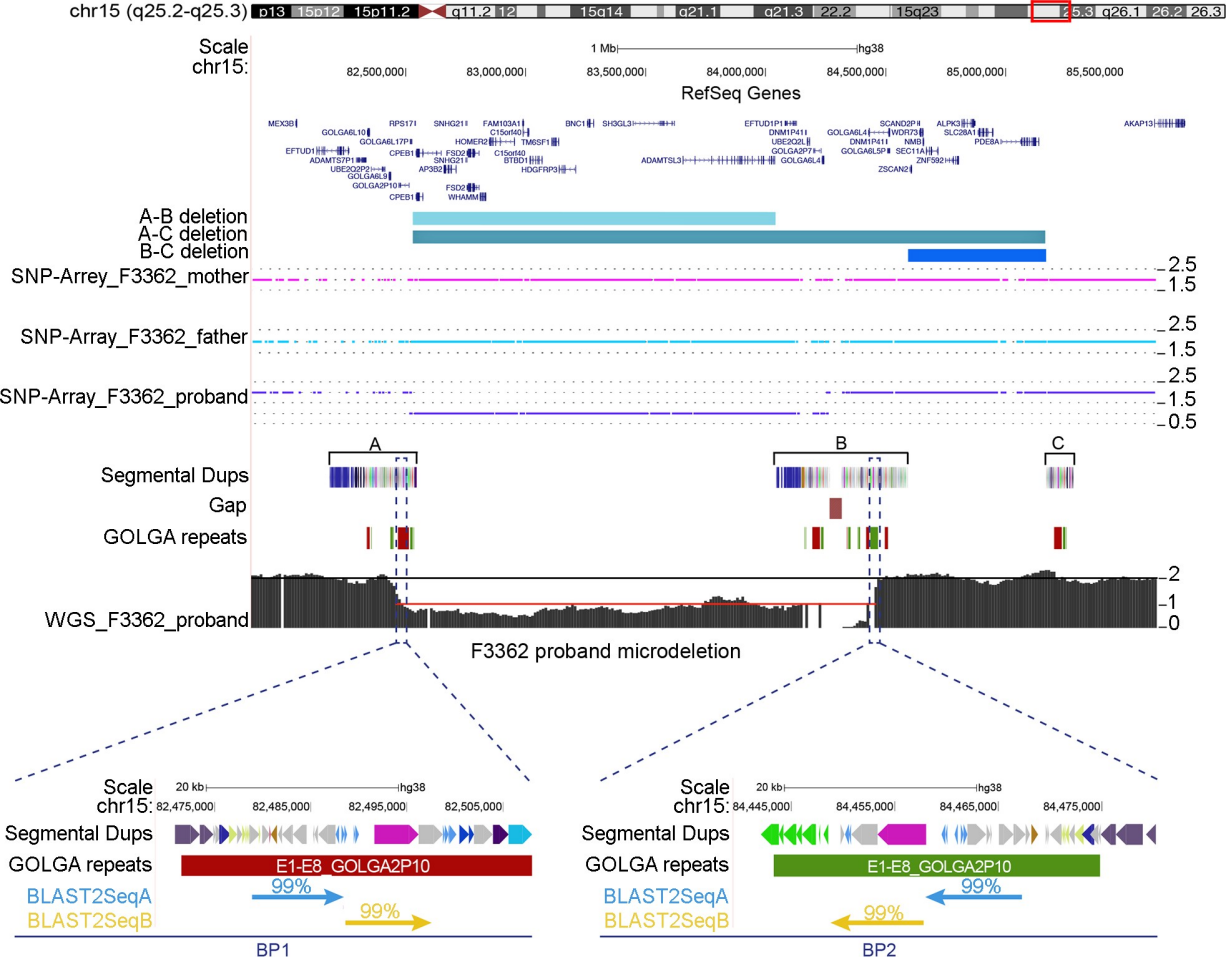
Deletions at the 15q25 region. Three different classes of microdeletions with breakpoints between segmental duplication blocks A-B, A-C and B-C are shown as colored boxes. Segmental duplications are shown with colored boxes. *GOLGA* repeat locations from the blat analysis of *GOLGA2P10* and *GOLGA6L5P* are depicted with green and red boxes mapping on plus or minus strand, respectively. SNP array and whole-genome sequencing (WGS) data of a patient with a 15q25 A-B deletion are shown. The SNP array highlights a copy number (CN) of 2 for the parents while the proband shows a CN of 1 for the deleted region. WGS shows a CN of 2 for the regions flanking the microdeletion (black line) and a CN of 1 (red line) for the deleted region. At the bottom of the figure are shown the results of a blast2Seq alignment between the two microdeletion breakpoint intervals. The two largest alignments of 9.4 kbp (blast2SeqA) and 9 kbp (blast2SeqB) with 99% similarity are shown with light blue and yellow arrows, respectively. *GOLGA2P10* repeats, which encompass the regions of high similarity at the breakpoints, are also shown in the zoom inset.

Three different classes of 15q25 microdeletions associated with developmental delay and intellectual disability have been described [19–24]. The microdeletions can be classified based on recurrent breakpoints, with breakpoints between duplication blocks A and B (A-B deletions), B and C (B-C deletions) and A and C (A-C deletions). The three blocks of segmental duplications that mediate the rearrangements are 350 kbp, 560 kbp and 115 kbp in size. Each block contains at least one copy of the *GOLGA* core duplicon (Fig 4; S1 Table).

Using single-nucleotide polymorphism (SNP) microarrays, we analyzed a patient with global developmental delay harboring a 15q25 deletion and mapped the disease-critical region to a 1.6 Mbp region spanned by segmental duplication blocks A and B (Fig 4). To refine the breakpoints with greater precision, we performed whole-genome sequencing (WGS) of the 15q25 deletion sample using Illumina HiSeq X Ten (150 bp PE reads; 26.2X coverage for whole chromosome 15) and aligned the sequences to the human reference. Using singly unique nucleotide (SUN) variants that allowed us to discriminate between the paralogous copies [39], we narrowed the deletion breakpoints to a 20 kbp (chr15:82478935–82498934) segment within duplication block A and a 101 kbp (chr15:84404774–84506312) segment within block B. Blast2seq analysis of the sequence mapping within the breakpoints intervals shows that the longest alignments with 99% similarity correspond to two sequences of 9.4 kbp (blast2SeqA) and 9 kbp (blast2SeqB), mapping at *GOLGA2P10* sequences, in inverted orientation (Fig 4; S1 File).

### Erosion of recombination suppression at 15q25 in great apes

Centromeres and their neighboring pericentromeric chromatin are well-established cold spots of meiotic crossover activity [3–5]. We hypothesized that inactivation of the ancestral centromere at 15q25 reduced the strength of recombination suppression at this locus, leading to higher rates of rearrangement and genome instability. Three lines of evidence support this interpretation. First, there are active meiotic recombination hotspots within the 15q25 region of the human genome [40]. Second, *GOLGA* repeats within the 15q25 region engage in frequent bouts of interlocus gene conversion [41]. Both of these observations point to homology-driven repair activity within the 15q25 region. Third, broad-scale recombination rates over the 15q25 region are elevated relative to the recombination levels of active centromeres and pericentromeric regions in the human genome. We observe a similar relaxation of recombination rate suppression in the inactivated 15q25 ancestral centromere region in bonobo, chimpanzee, and gorilla. We caution that recombination map quality is likely reduced across the structurally complex 15q25 locus, and the absence of informative centromeric markers precludes estimates of recombination rate within these gapped regions on the assembly. However, these qualitative findings suggest that the repositioning of the chromosome 15 centromere in the common ancestor of great apes weakened the recombination-suppressive environment that defines centromeres and set the stage for recurrent homology-driven rearrangements at the human 15q25 locus.

## Discussion

In this study, we sought to better understand the mechanisms leading to the genomic instability of the 15q25 locus by characterizing evolutionary and contemporary rearrangements by FISH, single-cell Strand-seq, PacBio SMRT sequencing, and Bionano optical mapping analyses. Chromosome 15q25 harbors a complex genomic region with three large blocks of segmental duplications containing several copies of the *GOLGA* core duplicon, previously shown to be involved in several recurrent pathogenic and evolutionary rearrangements on chromosome 15 [2, 12–14]. The middle block of segmental duplications contains a gap in the current release of the human reference assembly. Using SMRT technology, we sequenced and *de novo* assembled a tiling path of four BAC clones (657 kbp region) across this medically relevant region from the library of a hydatidiform mole and showed that the gap was flanked by two identical copies of *GOLGA* core duplicons that might have confounded mapping and assembly of the region. Alternatively, this might be a biological difference in structural haplotypes and/or copy number of *GOLGA* between human individuals.

Microarray analysis of 15q25 microdeletions in previous [19–24] and current studies refined breakpoint locations to segmental duplication blocks A, B and C; however, probe cross-hybridization prevented further narrowing of breakpoint locations within the duplications. All three segmental duplication clusters have multiple regions of high sequence identity—the most significant in direct orientation has 99% identity across 59 kbp. Here we performed WGS of a 15q25 microdeletion between duplication blocks A and B. SUN variant mapping allowed us to differentiate the segmental duplication paralogous copies [39] and revealed that the breakpoints map precisely to the *GOLGA* core duplicon sequences, which are organized in a palindromic configuration. Previous studies of many different chromosome 15 rearrangements, including several recurrent microdeletion/duplication syndromes and more complex rearrangements such as inverted duplications and triplications of chromosome 15 [13, 34], have shown that the breakpoints of all of these appear to coincide precisely with the location of the duplication family containing the *GOLGA* gene. An example is the 2 Mbp microdeletion encompassing the 15q13.3 region associated with intellectual disability, schizophrenia, autism and epilepsy [13]. WGS of two idiopathic autism patients carrying *de novo* 15q13.3 microdeletions showed that the two probands have different breakpoints but in both cases they map to *GOLGA* sequences [13].

Taken together these results suggest that despite the presence of large segmental duplications in direct orientation, known to be a predisposing factor for non-allelic homologous recombination (NAHR) leading to deletion/duplication events, palindromic *GOLGA* repeats seem to be preferential sites for NAHR promoting disease-related instability of chromosome 15. The presence of *GOLGA* core duplicons at multiple disease-associated rearrangements [13, 15–18] and evolutionary breakpoints [12–14] indicate the high level of genomic instability driven by these sequences.

**Fig 5:**
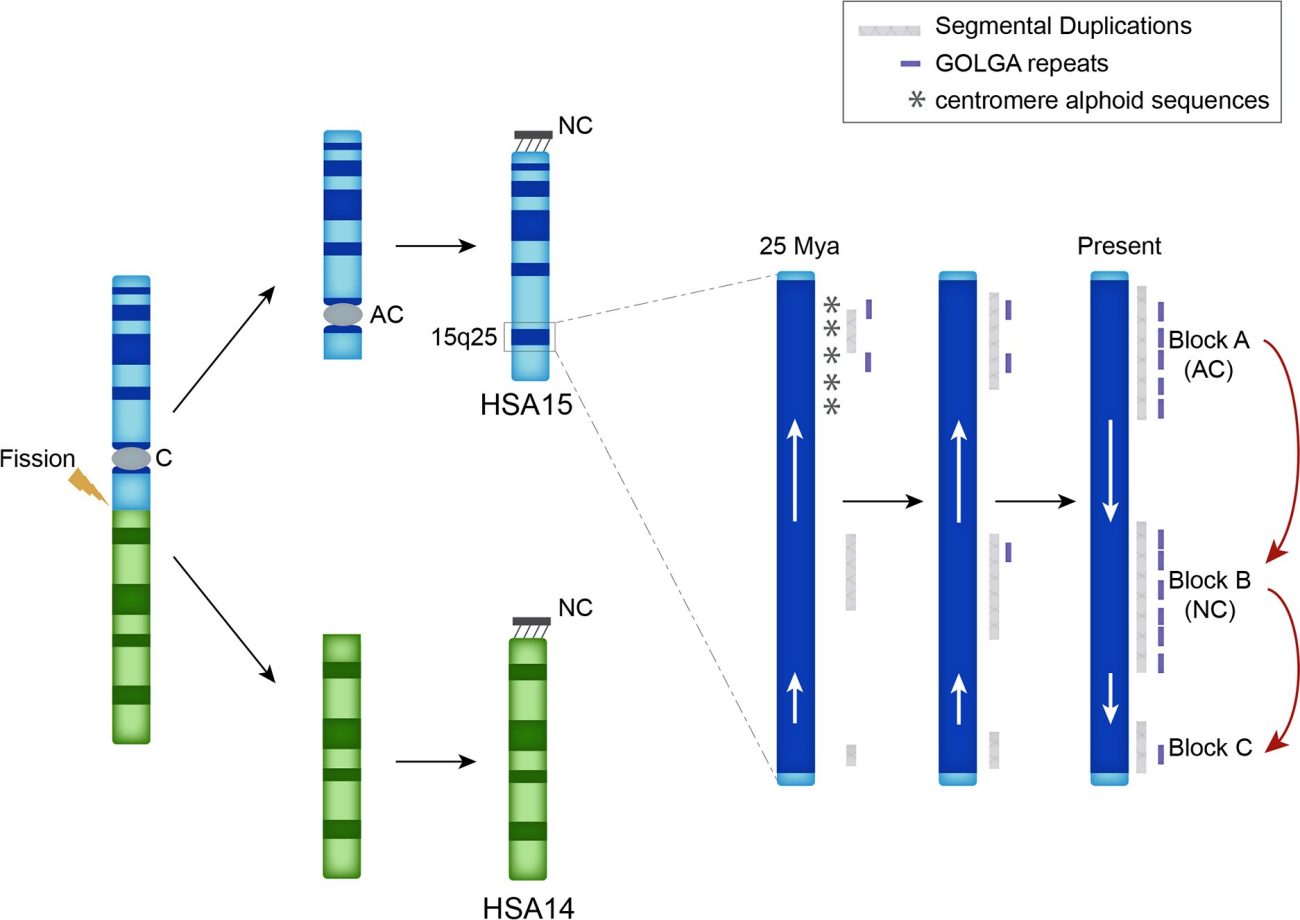
Human chromosome 15 evolution and *GOLGA* core elements dispersion. A fission event of an ancestral chromosome led to human and great ape chromosomes 14 (green ideogram) and 15 (blue ideogram). The zoomed-in view shows how the inactivation of the ancestral centromere after the fission event released the recombination constraints typical of pericentromeric regions leading to two inversions, shown with the white arrows, which resulted in a dispersal of *GOLGA* repeats (purple blocks) and segmental duplications (gray bars). AC, ancestral centromere. NC, neocentromere.

The chromosome 15q25 locus approximates the position of the ancestral centromere, which became inactivated about 25 million years ago [2]. This inactivation followed a noncentromeric chromosomal fission of an ancestral chromosome that gave rise to human and great apes chromosomes 14 and 15 [2]. The duplications flanking the ancestral centromere were formed within a pericentromeric context where recombination was almost absent [3–5]. Our recombination and evolutionary analyses support the hypothesis that following inactivation of the ancestral centromere, the constraint against recombination in this area was relaxed, rendering the locus permissive to NAHR-mediated rearrangements. We speculate that such events ultimately led to two local inversions specific to humans and African great apes. These inversions may have ultimately helped to disperse the *GOLGA* core elements that are now mediating pathogenic microdeletions (Fig 5). To test this hypothesis, we performed a BLAT analysis on the latest releases of the chimpanzee, gorilla, orangutan and macaque genome assemblies and found that human has nine copies of the core duplicon in excess compared to macaque.

The distal inversion is polymorphic within the chimpanzee population (39% allele frequency), while all the other species are configured in the opposite orientation compared to human suggesting that the inversion occurred in the human–chimpanzee ancestor and is highly polymorphic in chimpanzee. These findings are strikingly reminiscent of the 17q21.31 chromosome inversion [42], which is flanked by *LRRC37* core duplications on either side of the inversion region and is highly polymorphic in multiple species, especially in chimpanzee (56% allele frequency).

The proximal inversion is 1.5 Mbp in size and likely occurred in the human–African great ape ancestor. The orthologous region in chimpanzee, however, is in the opposite orientation as that of human, suggesting that the region either flipped back to the ancestral orientation in the chimpanzee lineage or the chimpanzee configuration may represent incomplete lineage sorting of an ancestral state. FISH and Strand-seq analyses of 44 human samples show that this inversion is still polymorphic in humans, with a minor allele frequency of 1.14%. The inversion corresponds to the exact same region that is deleted in patients with intellectual disability, suggesting that the inversion may represent a premutation state to pathogenic rearrangements, as previously observed for other regions of the genome, such as the 17q21.31, 7q11.23 and 8p23.1 loci [42–44]. The inversion could change the orientation and composition of *GOLGA* repeats (depending on breakpoints), and this could impact the likelihood of a recombination event generating a deletion.

Interestingly, chromosome 15q25 is also one of the hotspots of neocentromere appearance in clinical cases [45]. The majority of neocentromeres reported in clinical samples rescue acentric chromosome fragments associated with duplications or chromosomal rearrangements found in patients with developmental disabilities [46]. From a literature review we identified several recurrent rearrangements, consisting of tetrasomies from 15q25→qter due to analphoid chromosomal markers with a neocentromere at 15q25 (S5A Fig) [2, 25–29, 46–63]. Ventura and colleagues performed detailed mapping of the ancestral centromere as well as a neocentromere mapping to chromosome 15q25 and established that the ancestral centromere and the neocentromere map to two different clusters of segmental duplications separated by a 1.5 Mbp single-copy region [2]. Notably, we show that this region corresponds exactly to the 1.5 Mbp proximal inversion that occurred in the human–African great ape ancestor after inactivation of the ancestral centromere. Following inactivation, an increased frequency of ectopic rearrangements at 15q25 might have resulted in evolutionary inversions that led to a duplicative transposition of *GOLGA* core elements from one breakpoint to the other (from segmental duplication block A to B).

The existence of human individuals heterozygous for the proximal inversion led us to hypothesize that inversions may be a driving force in the formation of 15q25 neocentric invdup marker chromosomes. FISH mapping of the breakpoints of invdup markers from both 8p and 15q suggests that between the two duplicated symmetrical arms is found an unduplicated region containing proximal sequences contiguous with one of the arms (S5B Fig) [2, 44]. Such an arrangement is consistent with meiotic recombination between chromosomes that are heterozygous for a polymorphic inversion flanked by inverted segmental duplications. During cell division, asynapsis at the inverted region may promote the refolding of one chromosome onto itself, allowing intrachromatid synapsis and NAHR between two *GOLGA* repeats (S5C Fig). This would favor the formation of the 15q25→qter inverted-duplicated chromosomal markers, similar to what has been previously shown for the 8p23.1 inversion polymorphism [44]. In conclusion, our findings highlight the intimate relationship between inversions and core duplicons and reinforce the hypothesis that *GOLGA* repeats played a fundamental role in shaping the architecture of chromosome 15 in humans and great apes and continue to predispose it to disease-causing rearrangements.

In conclusion, we propose the following model. Core duplicons in 15q25 were formed within a pericentromeric context, and sequences in the same region have continued to undergo sequence exchange/duplication within the human lineage long after the centromere became inactivated about 25 million years ago. This had significant implications for genomic stability in the region. It is highly likely that following inactivation of the ancestral centromere, the constraint against recombination in this area was relaxed. This would have increased the frequency of ectopic rearrangements, accelerating the dispersal of the linked *GOLGA* duplicons and leading to two local inversions specific to humans and African great apes. Our findings highlight the intimate relationship between inversions and core duplicons and reinforce the hypothesis that *GOLGA* repeats played a fundamental role in shaping the architecture of chromosome 15 in humans and great apes and continue to predispose it to disease-causing rearrangements.

## Material and methods

### Ethics statement

The study was approved (Prot. 117CE;6/11/2017) by the local Ethics committee of the IRCCS "Casa Sollievo della Sofferenza" Hospital, San Giovanni Rotondo (FG). Written, informed consent was received.

### FISH analysis

Interphase nuclei were obtained from lymphoblast and fibroblast cell lines from 22 human HapMap individuals (Coriell Cell Repository, Camden, NJ, USA), eight chimpanzees (*Pan troglodytes*), four orangutans (*Pongo pygmaeus*), four gorillas (*Gorilla gorilla*) and one macaque (*Macaca mulatta*) (S2 Table). FISH experiments were performed using human fosmid (n = 6) clones (S6 Table) directly labeled by nick-translation with Cy3-dUTP (Perkin-Elmer), Cy5-dUTP (Perkin-Elmer) and fluorescein-dUTP (Enzo) as described by Lichter et al. [64], with minor modifications. Briefly, 300 ng of labeled probe were used for the FISH experiments; hybridization was performed at 37°C in 2xSSC, 50% (v/v) formamide, 10% (w/v) dextran sulphate and 3 mg sonicated salmon sperm DNA, in a volume of 10 mL. Posthybridization washing was at 60°C in 0.1xSSC (three times, high stringency, for hybridizations on human, chimpanzee, gorilla and orangutan) or at 37°C in 2xSSC and 42°C in 2xSSC, 50% formamide (three times each, low stringency, for hybridizations on macaque). Nuclei were simultaneously DAPI stained. Digital images were obtained using a Leica DMRXA2 epifluorescence microscope equipped with a cooled CCD camera (Princeton Instruments). DAPI, Cy3, Cy5 and fluorescein fluorescence signals, detected with specific filters, were recorded separately as grayscale images. Pseudocoloring and merging of images were performed using Adobe Photoshop software. Proximal and distal inversions were interrogated using two probes within the putative inversion region and a reference probe outside, as previously described [37].

### SNP array assay

SNP array-based copy number variant (CNV) analysis was performed on genomic DNA extracted from peripheral blood lymphocytes of the patient and parents, after obtaining written informed consent, using the CytoScan HD Array (Affymetrix, Santa Clara, CA, USA) as previously described [65]. Data analysis was performed using the Chromosome Analysis Suite software version 3.1 (Affymetrix, Santa Clara, CA, USA). A CNV was validated if at least 25 contiguous probes showed an abnormal log2 ratio. The clinical significance of each CNV detected was assessed by comparison with public databases such as the Database of Genomic Variants (DGV; available online at: http://dgv.tcag.ca/), DECIPHER (https://decipher.sanger.ac.uk/), and ClinVar (https://www.ncbi.nlm.nih.gov/clinvar/). We also checked an internal database of 3,500 patients studied by SNP array in our laboratory since 2010 with a diagnosis of syndromic/non-syndromic neurodevelopmental disorders. Finally, to predict the pathogenic role of the identified microdeletions/microduplications, we followed the American College of Medical Genetics guidelines [66].

### Whole-genome sequencing of a 15q25 microdeletion sample

Read-depth profiles were generated by extracting the first 36 bp of each read from a BWA-MEM aligned BAM file and aligning these reads to the hg38 genome at all possible positions using mrsFAST-Ultra [67]. Read-depth profiles were converted to copy number estimates at edit distances of 2 and 0 to define total and locus-specific (SUN) copy number estimates at ~93% of confidence, as described in Sudmant et al. 2010 [39]. Coverage for chromosome 15 is 26.2X for the full reads and ~6.3X for the first 36mer. Sequence read-depth corresponding to SUN variants was then used to refine the microdeletion breakpoints as previously described [39].

### Strand-seq data analysis

The inversion status of Strand-seq libraries generated from a pooled cord blood sample comprising 47 unrelated donors (described in detail in: [30]) was assessed at the 15q25 locus. Briefly, Strand-seq sequence data were aligned to GRCh37/hg19, BED-formatted for upload to the UCSC Genome Browser, and analyzed using the open-source ‘Invert.R’ software (https://sourceforge.net/projects/strandseq-invertr/). Only cells that inherited chromosome 15 in the WW (W, Watson; reverse or minus strand) or CC (C, Crick; forward or plus strand) were analyzed (n = 22) to ensure homozygous inversions were fully captured. Libraries were tested for a segmental change in strand orientation at the putative inversion loci (coordinates lifted to GRCh37/hg19; proximal inversion at chr15:83202890–84714735 and distal inversion at chr15:85139055–85713003), and genotypes were confirmed from the Invert.R results [30].

### *GOLGA* copy number analysis

To estimate the copy number of *GOLGA* repeats in all the tested species we performed a blat analysis. We downloaded *GOLGA2P10* and *GOLGA6L5P* exon sequences from the UCSC Genome Browser and used the blat tool to compare them with the human reference (GRCh38/hg38). We also performed the same analysis for chimpanzee (Clint_PTRv2/panTro6), gorilla (gorGor4.1/gorGor4), orangutan (Susie_PABv2/ponAbe3), and macaque (BCM Mmul_8.0.1/rheMac8).

We then analyzed the copy number of *GOLGA* in 15q25 locus using PacBio-based assemblies of human CHM1 (https://www.ncbi.nlm.nih.gov/assembly/GCA_001297185.1), chimpanzee (https://www.ncbi.nlm.nih.gov/assembly/GCF_002880755.1), gorilla (https://www.ncbi.nlm.nih.gov/assembly/GCA_900006655.3), and orangutan (https://www.ncbi.nlm.nih.gov/assembly/GCF_002880775.1). Contigs from PacBio assemblies were aligned to the human reference using Mashmap 2.05 with default parameters. Three filtering steps were then applied to the alignments. First, alignments were filtered such that contigs were only mapped to one location in GRCh38. Second, remaining alignments were intersected with the 24 regions found in the BLAT analysis using BEDTools6. Finally, these intersections were filtered to only those with at least 90% overlap with one of the 24 defined *GOLGA* regions. After these steps, the *GOLGA* copy number was estimated by counting the number of *GOLGA* regions that were intersected by each primate assembly.

### PacBio SMRT clone sequencing and assembly

DNA was isolated from CH17, CH251 and CH276 BAC clones (S3 Table) as previously described [68]. PacBio (Pacific Biosciences, Inc., Menlo Park, CA, USA) SMRTbell libraries were prepared and sequenced using RS II P6-C4 chemistry. We performed *de novo* assembly of pooled BAC inserts (5–6 BACs per pool) using the Canu assembler [69] followed by consensus calling using Quiver [68]. PacBio assemblies were reviewed for misassembly by sequencing to a minimum coverage depth of 200X and visualizing read depth of PacBio reads in Parasight (http://eichlerlab.gs.washington.edu/jeff/parasight/index.html) using coverage summaries generated during the resequencing protocol [68]. As a final validation, we mapped publically available BAC end sequences to high-quality finished clone inserts to confirm order and orientation. Human, chimpanzee, orangutan and macaque assemblies, including PacBio sequenced clones from CH17, CH251, CH276 and CH250 BAC libraries, were assembled with Sequencher and compared to the human reference genome using Miropeats [70] and BLAST [71]. Duplication analysis using whole-genome shotgun sequence detection (WSSD) was performed as previously described [72].

### Bionano Genomics optical mapping

High molecular weight DNA from one chimpanzee (Clint), one gorilla (Kamilah), and one orangutan (Susie) were used to construct Bionano optical maps (S2 Table). The chimpanzee and orangutan maps were constructed as previously described by Kronenberg et al. [73]. To label the chimpanzee and orangutan genomes, two different enzymes were used to ensure contiguous coverage. The two labelling enzymes used (Nt.BspQI and Nb.BssSI) are nickases, and each enzyme makes single-stranded nicks at specific recognition motifs along the genomes. However, whenever two nick sites in opposite strands are in close proximity, double-stranded breaks would be created in the DNA. Such double-stranded breaks—also known as fragile sites—would create permanent disruptions in the DNA molecules and the assembled contigs. Therefore, to bridge these fragile sites, in a separate labelling experiment, a second enzyme that recognizes a different motif site was used. The gorilla maps were constructed using DLE-1 non-nicking enzyme, which is a newer enzyme that directly labels its recognition sites without creating any nicks. Thus, for that experiment, no fragile sites were created, and we achieved continuous coverage across the genome. Briefly, labelling and staining of the DNA were performed according to a protocol developed by Bionano Genomics. Labelling was performed by incubating 750 ng genomic DNA with 1X DLE-1 Enzyme (Bionano Genomics) for 2 hours at 37°C, followed by 20 minutes at 70°C, in the presence of 1X DL-Green and 1X Direct Labelling Enzyme (DLE-1) Buffer. Following proteinase K digestion and DL-Green cleanup, the labelled DNA was mixed with 1X Flow Buffer, in the presence of 1X DTT, and left to incubate overnight at 4°C. Staining was performed by adding 3.2 μl of a DNA stain solution for every 300 ng of pre-stained DNA and incubating at room temperature for at least two hours before loading onto the Bionano Chip. Loading of the chip and running of the Bionano Genomics Saphyr System were all performed according to the Saphyr System User Guide (https://bionanogenomics.com/support-page/saphyr-system/).

### Recombination rate data and analysis

Fine-scale recombination rates for human, chimpanzee, bonobo, and gorilla were obtained from previously published sources [74, 75]. Recombination rates were averaged over 500 kbp windows and plotted as a function of distance to active, annotated centromeres on hg38 and the ancestral centromere at 15q25. Recombination data were smoothed using locally weighted smoothing with α = 0.05 for ease of visualization. The coordinates of the ancestral 15q25 centromere were delineated by clones RP11-152F13 and RP11-635O8 [2]. The positions of known recombination hotspots [40] and documented sites of interlocus gene conversion [41] were used to substantiate evidence for homology-driven repair activity at 15q25.

### Data access

Sequencing data from Illumina HiSeq X Ten can be found at the Sequence Read Archive (SRA) under BioProject ID PRJNA493749, as BAM file (GRCh38/hg38). PacBio SMRT sequences of CH17 clones can be found under BioProject ID PRJNA514724. Complete sequences of primate BAC clones (S3 Table) can be found as NC_036894.1, NC_036918.1, AC275844.1, AC212984.3, AC210775.3 and AC211297.2.

## Supporting information

S1 Fig PacBio SMRT sequencing of CH17 BAC clones across the gap region. Shown is a Miropeats comparison of a contig obtained from PacBio SMRT sequencing of four CH17 BAC clones and the hg38 human reference. Results due to segmental duplications are shown with gray lines. Black lines connect matching segments between the CH17 contig (bottom) and the reference genome (top), gray lines connect segmental duplications, and red lines connect the region spanning the gap, present once in the CH17 contig and twice in the reference genome. Segmental duplication colors show the ancestral origins of duplications based on comparison with mammalian groups assigned by DupMasker [76]. *GOLGA* repeats mapping on the plus and minus strands are depicted with green and red boxes, respectively.
(TIF)

S2 Fig PacBio SMRT sequencing of primate BAC clones. (A) Comparison between human and orangutan duplication blocks. Duplication blocks B and C in human are shown within orange and green boxes, respectively. The purple box indicates the B/C duplication block hybrid in orangutan. Miropeats comparison of sequenced clones mapping at the duplication blocks in human and orangutan genomes are shown. Black lines connect matching segments between human and orangutan. Green and purple lines represent sequence in inverted orientation between the two species, with the green lines showing the proximal breakpoint of the 600 kbp distal inversion. Differences in *GOLGA* repeat content between human and orangutan are also shown. Segmental duplication colors show the ancestral origins of duplications based on comparison with mammalian groups assigned by DupMasker [76]. (B) Miropeats comparison of the B/C hybrid block region in orangutan and macaque. Black lines connect matching segments between human and macaque while red lines indicate sequences in inverted orientation within the B/C hybrid block in macaque relative to orangutan. *GOLGA* repeats in orangutan and macaque are shown. (C) Miropeats comparison of a chimpanzee sequenced clone mapping at the C duplication block in human and chimpanzee genomes. Black lines connect matching segments between human and chimpanzee. *GOLGA* repeats in chimpanzee and human are also shown.
(TIF)

S3 Fig *GOLGA2P10* and *GOLGA6L5P* BLAT results. Results of BLAT analysis against the three segmental duplication blocks at the 15q25 locus are shown. Gray and light blue bars represent, respectively, *GOLGA2P10* and *GOLGA6L5P* exon BLAT results with white arrowheads inside the bars showing the orientation of the repeats. Segmental duplications and RefSeq genes (curated subset) are also shown.
(TIF)

S4 Fig Recombination rates across human, chimpanzee, bonobo, and gorilla. Broad-scale recombination rates for human, chimpanzee, bonobo, and gorilla autosomes (grey lines) plotted as a function of distance from active, annotated centromeres (red dashed line) on hg38. For chromosome 15 (black line), recombination rates are also plotted as a function of distance from the ancestral 15q25 centromere (red dashed line). Recombination rates were averaged over 500kbp windows. For each figure panel, lines are zero-centered on the autosomal centromere midpoints or the ancestral 15q25 centromere locus (red dashed line). Although recombination rates are reduced in the vicinity of the ancestral 15q25 centromere, suppression is not as strong as observed for active centromeres.
(TIF)

S5 Fig Mechanism of marker chromosomes formation. (A) Colored bars show microdeletions and inverted-duplicated supernumerary marker chromosomes (SMCs) involving the 15q25 region, previously characterized either by array CGH or FISH analyses. Two sets of marker chromosomes have been described up to now, consisting of an inverted-duplicated segment either between duplication block B and 15qter (Set 1) or between duplication block C and 15qter (Set 2). (B) On the left is depicted the marker chromosome characterized by Ventura and colleagues. Two duplicated segments (external blue arrowheads) are separated by a stretch of single-copy sequence. NC, neocentromere. On the right, two chromosome 15 homologs are shown with and without the proximal inversion. The orientation of the distal and proximal regions are shown by colored arrowheads, in red for inverted orientation and in green for direct orientation. (C) Mechanisms leading to the formation of marker chromosomes at 15q25. During cell division, asynapsis at the inverted region promotes the refolding of either the direct or inverted chromosome onto itself, allowing intrachromatid synapsis and NAHR between *GOLGA* repeats mapping at segmental duplication blocks A, B and C. Unequal recombination between different blocks leads to the formation of SMCs of different sizes, with the unique region corresponding to the proximal and/or distal 15q25 regions, and a duplication (blue arrowheads) of the terminal part of the q arm. The SMC on the right side of the panel corresponds to the one described by Ventura and colleagues.
(TIF)

S1 File Blast2Seq alignment results. (PDF)

S1 Table BLAT results of *GOLGA2P10* and *GOLGA6L5P* in human and nonhuman primate reference genomes. (XLSX)

S2 Table Inversion analyses in human and nonhuman primate individuals. (XLSX)

S3 Table PacBio or Sanger sequenced clones. (XLSX)

S4 Table BLAT results of *GOLGA2P10* and *GOLGA6L5P* in human and nonhuman primate PacBio assemblies. (XLSX)

S5 Table Multiple regions of high sequence identity between three segmental duplication blocks. (XLSX)

S6 Table Fosmid clones used for FISH assays. (XLSX)
