# Supplementary figures and images for: Genomic inversions and GOLGA core duplicons underlie disease instability at the 15q25 locus
Source: PLoS Genet. 2019 Mar 27;15(3):e1008075. doi: 10.1371/journal.pgen.1008075 (PMC6436712; doi:10.1371/journal.pgen.1008075)

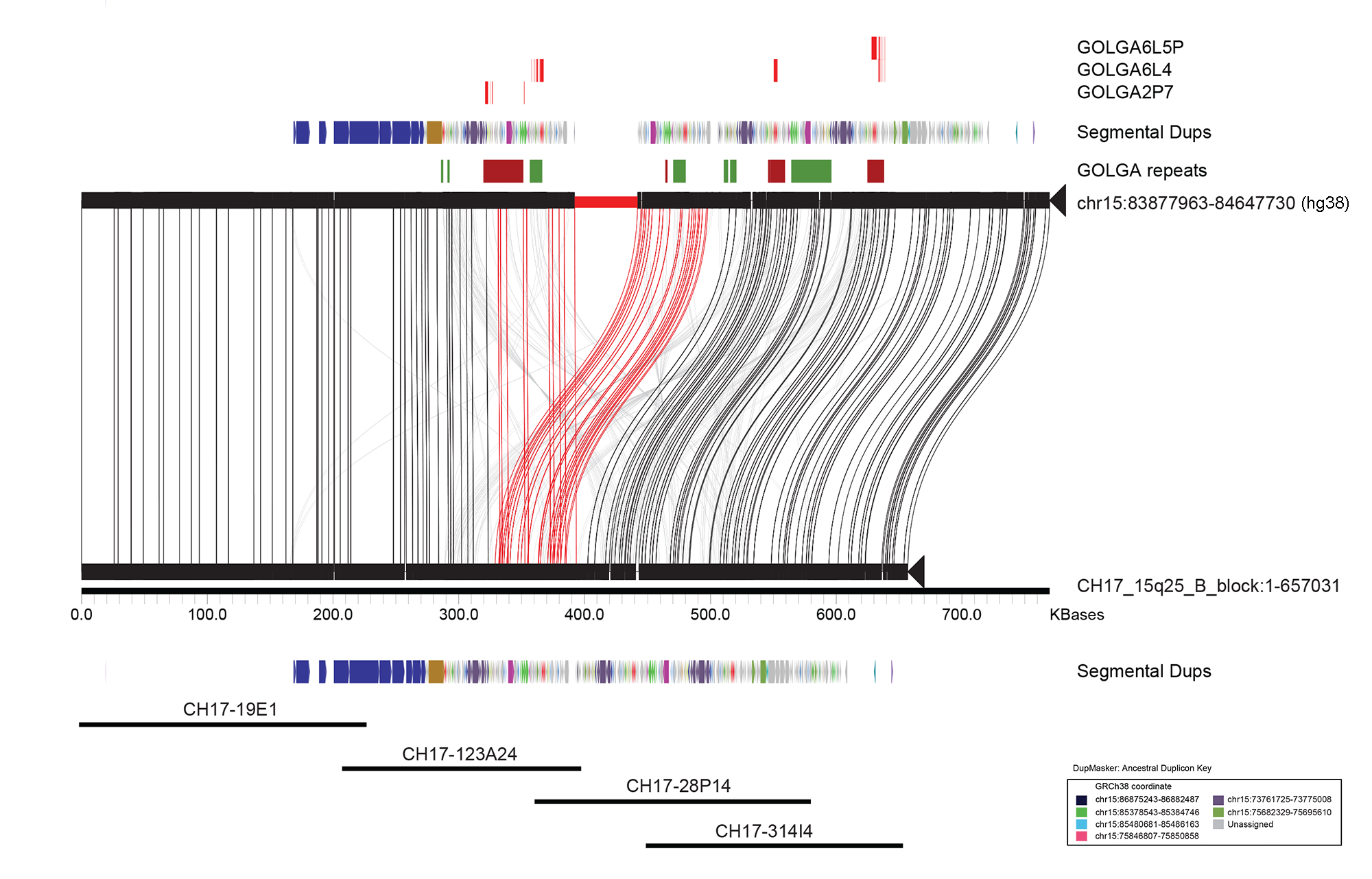

Supplement: S1 Fig — Shown is a Miropeats comparison of a contig obtained from PacBio SMRT sequencing of four CH17 BAC clones and the hg38 human reference. Results due to segmental duplications are shown with gray lines. Black lines connect matching segments between the CH17 contig (bottom) and the reference genome (top), gray lines connect segmental duplications, and red lines connect the region spanning the gap, present once in the CH17 contig and twice in the reference genome. Segmental duplication colors show the ancestral origins of duplications based on comparison with mammalian groups assigned by DupMasker [76]. GOLGA repeats mapping on the plus and minus strands are depicted with green and red boxes, respectively. (TIF) [file pgen.1008075.s001.tif]

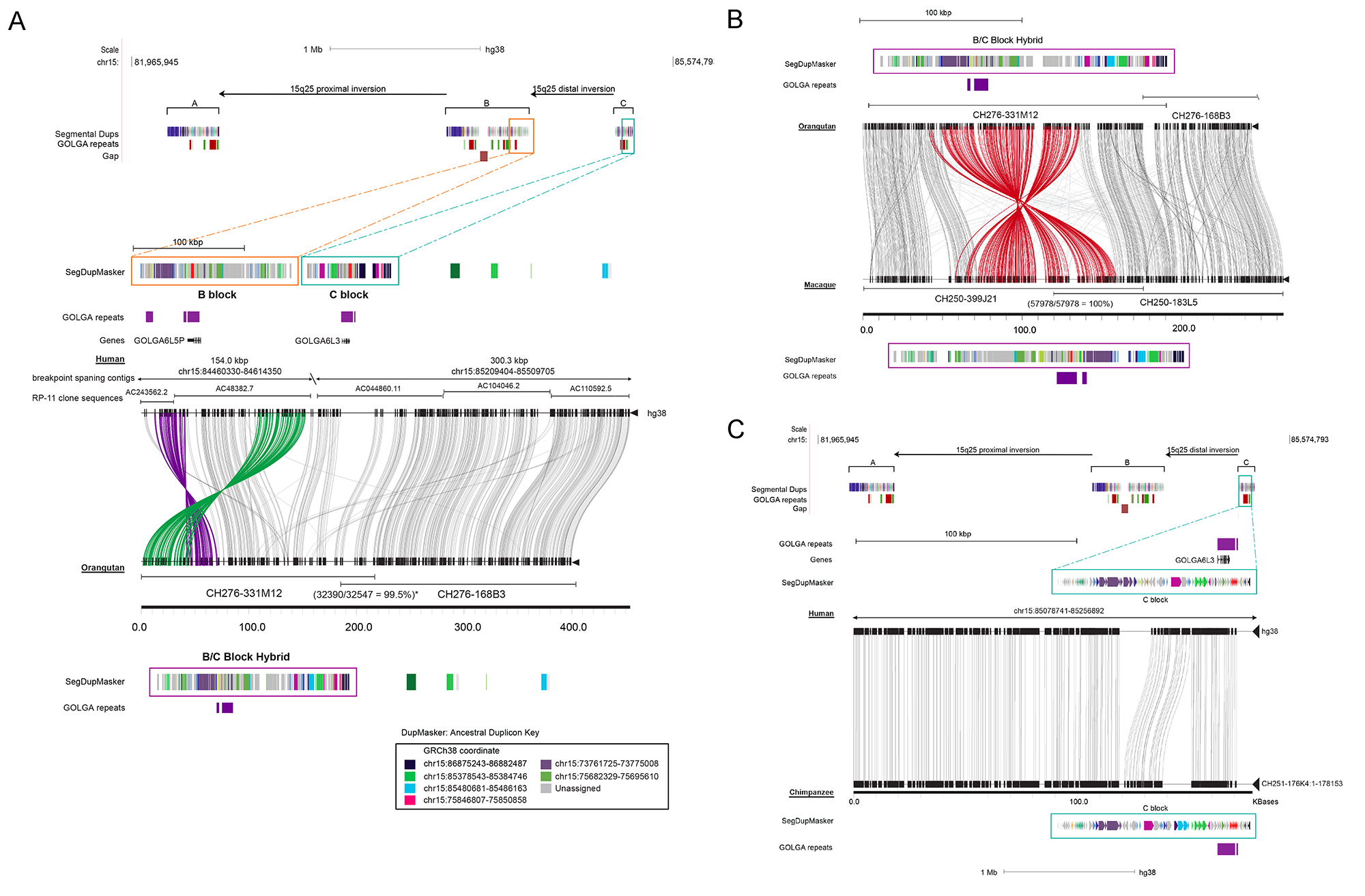

Supplement: S2 Fig — (A) Comparison between human and orangutan duplication blocks. Duplication blocks B and C in human are shown within orange and green boxes, respectively. The purple box indicates the B/C duplication block hybrid in orangutan. Miropeats comparison of sequenced clones mapping at the duplication blocks in human and orangutan genomes are shown. Black lines connect matching segments between human and orangutan. Green and purple lines represent sequence in inverted orientation between the two species, with the green lines showing the proximal breakpoint of the 600 kbp distal inversion. Differences in GOLGA repeat content between human and orangutan are also shown. Segmental duplication colors show the ancestral origins of duplications based on comparison with mammalian groups assigned by DupMasker [76]. (B) Miropeats comparison of the B/C hybrid block region in orangutan and macaque. Black lines connect matching segments between human and macaque while red lines indicate sequences in inverted orientation within the B/C hybrid block in macaque relative to orangutan. GOLGA repeats in orangutan and macaque are shown. (C) Miropeats comparison of a chimpanzee sequenced clone mapping at the C duplication block in human and chimpanzee genomes. Black lines connect matching segments between human and chimpanzee. GOLGA repeats in chimpanzee and human are also shown. (TIF) [file pgen.1008075.s002.tif]

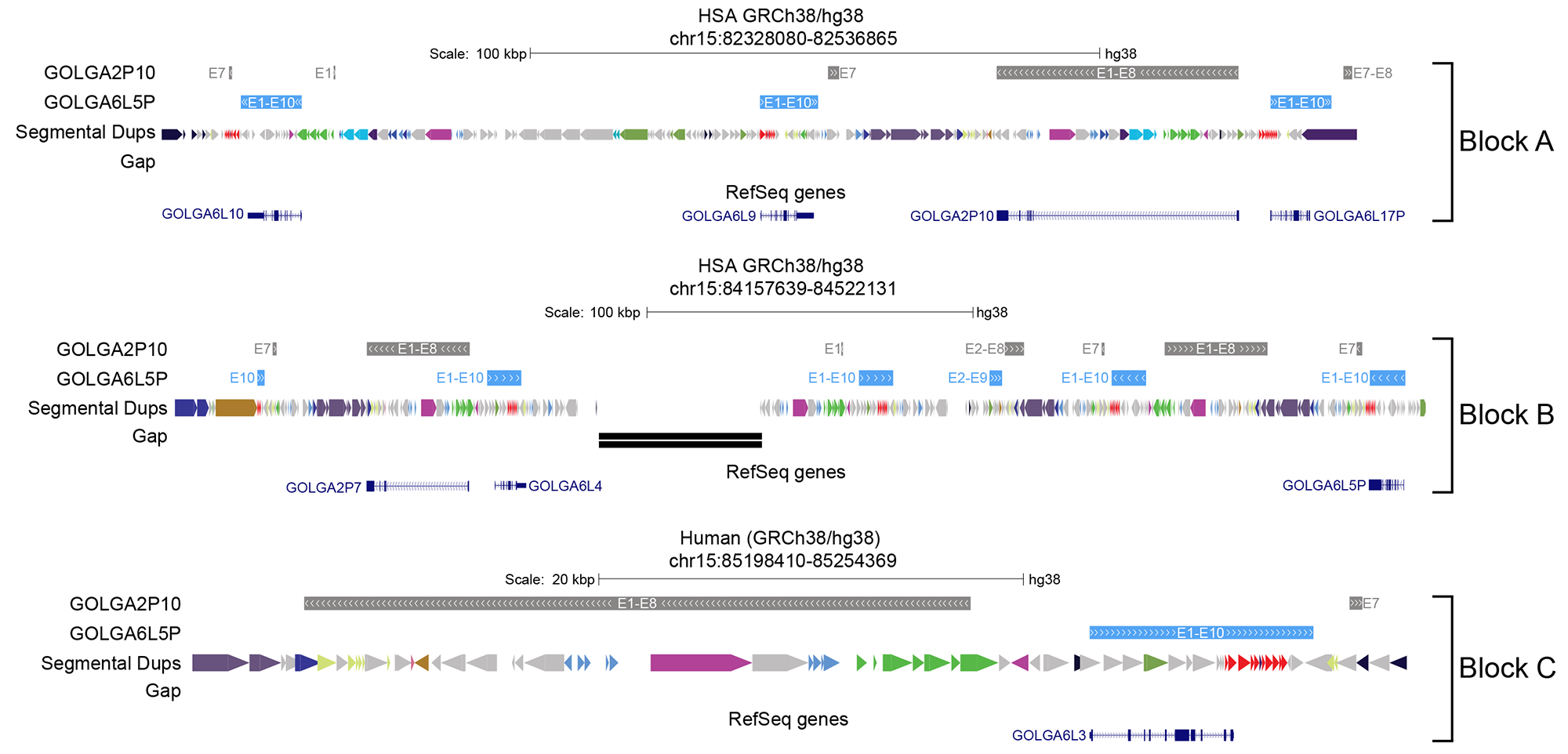

Supplement: S3 Fig — Results of BLAT analysis against the three segmental duplication blocks at the 15q25 locus are shown. Gray and light blue bars represent, respectively, GOLGA2P10 and GOLGA6L5P exon BLAT results with white arrowheads inside the bars showing the orientation of the repeats. Segmental duplications and RefSeq genes (curated subset) are also shown. (TIF) [file pgen.1008075.s003.tif]

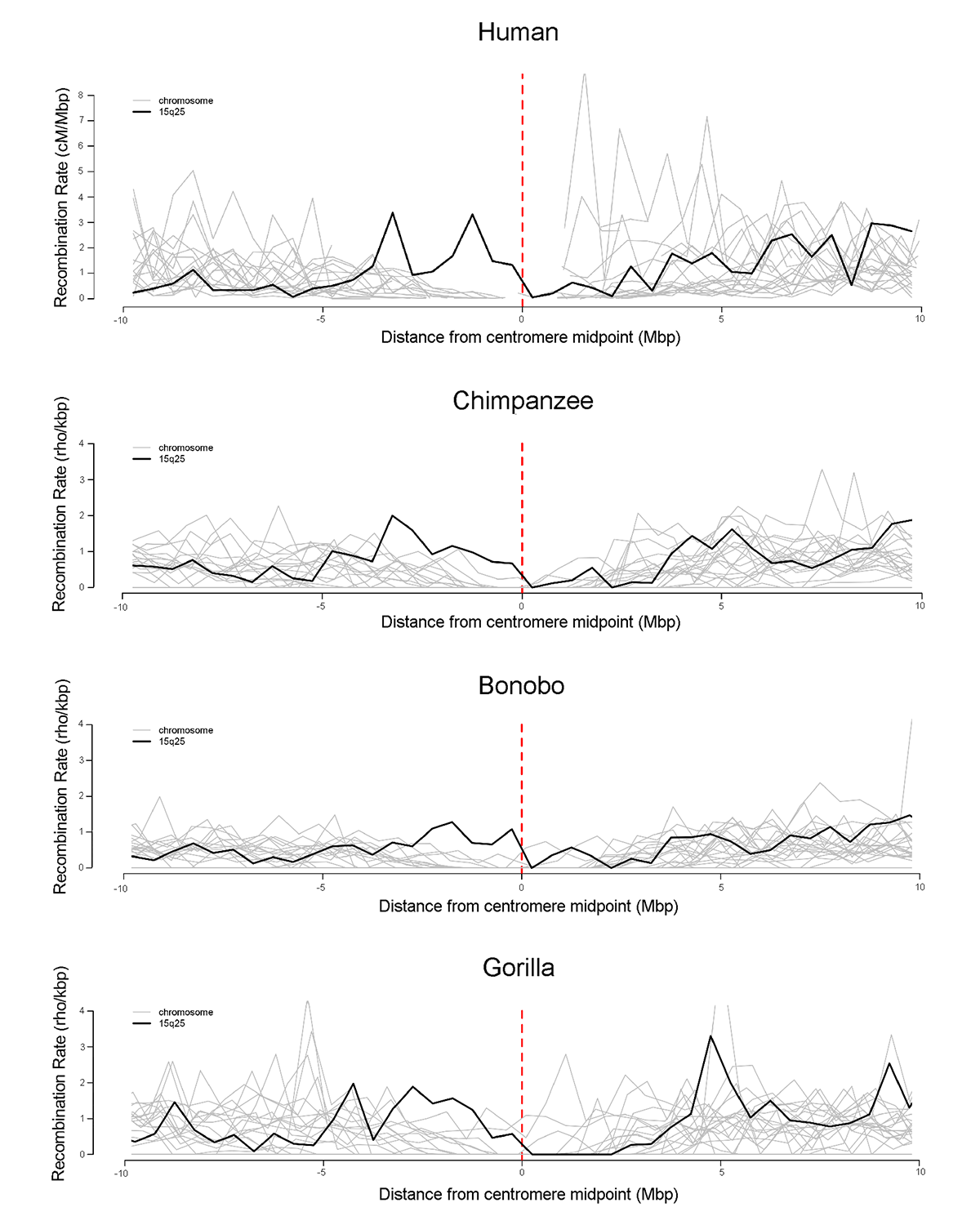

Supplement: S4 Fig — Broad-scale recombination rates for human, chimpanzee, bonobo, and gorilla autosomes (grey lines) plotted as a function of distance from active, annotated centromeres (red dashed line) on hg38. For chromosome 15 (black line), recombination rates are also plotted as a function of distance from the ancestral 15q25 centromere (red dashed line). Recombination rates were averaged over 500kbp windows. For each figure panel, lines are zero-centered on the autosomal centromere midpoints or the ancestral 15q25 centromere locus (red dashed line). Although recombination rates are reduced in the vicinity of the ancestral 15q25 centromere, suppression is not as strong as observed for active centromeres. (TIF) [file pgen.1008075.s004.tif]

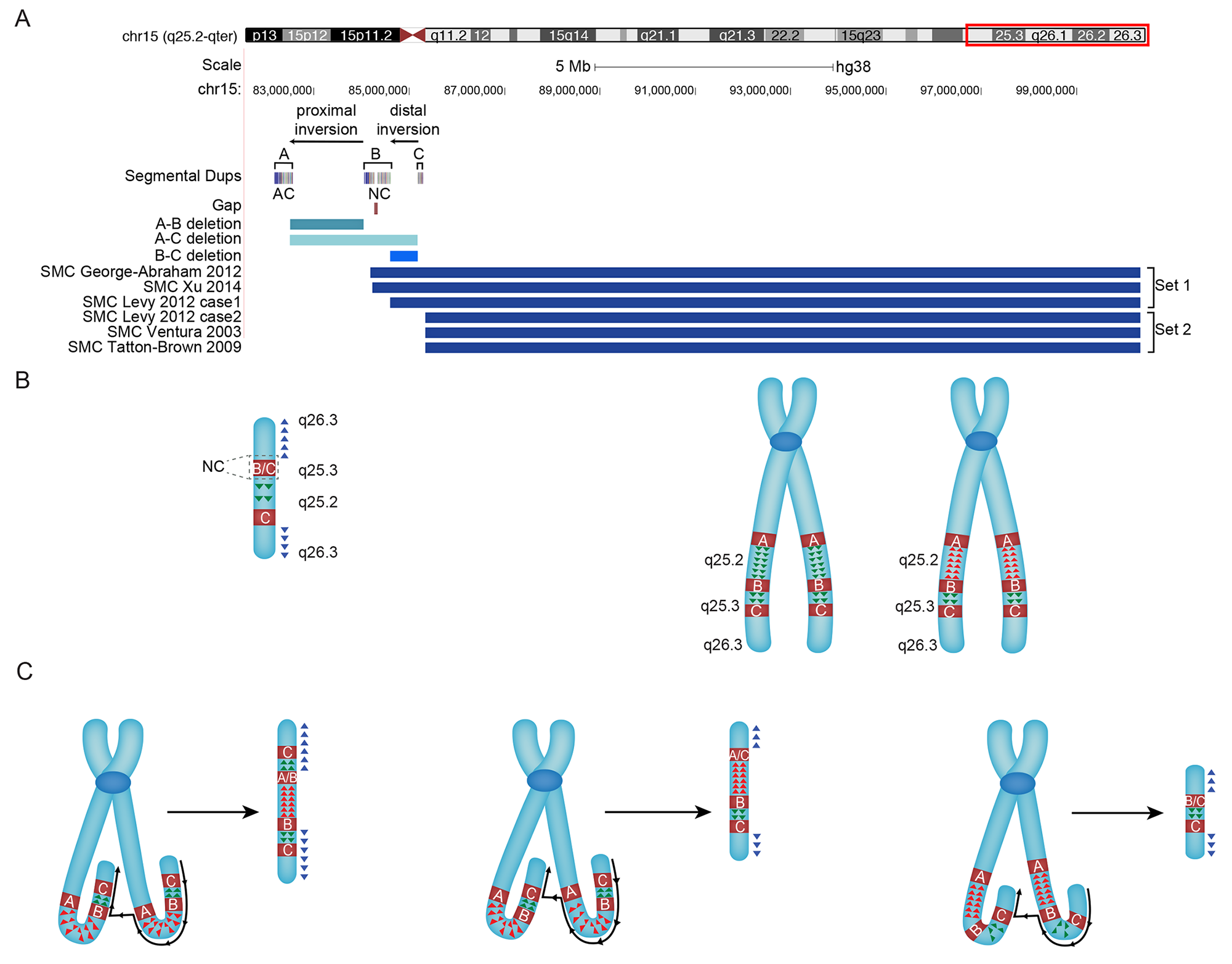

Supplement: S5 Fig — (A) Colored bars show microdeletions and inverted-duplicated supernumerary marker chromosomes (SMCs) involving the 15q25 region, previously characterized either by array CGH or FISH analyses. Two sets of marker chromosomes have been described up to now, consisting of an inverted-duplicated segment either between duplication block B and 15qter (Set 1) or between duplication block C and 15qter (Set 2). (B) On the left is depicted the marker chromosome characterized by Ventura and colleagues. Two duplicated segments (external blue arrowheads) are separated by a stretch of single-copy sequence. NC, neocentromere. On the right, two chromosome 15 homologs are shown with and without the proximal inversion. The orientation of the distal and proximal regions are shown by colored arrowheads, in red for inverted orientation and in green for direct orientation. (C) Mechanisms leading to the formation of marker chromosomes at 15q25. During cell division, asynapsis at the inverted region promotes the refolding of either the direct or inverted chromosome onto itself, allowing intrachromatid synapsis and NAHR between GOLGA repeats mapping at segmental duplication blocks A, B and C. Unequal recombination between different blocks leads to the formation of SMCs of different sizes, with the unique region corresponding to the proximal and/or distal 15q25 regions, and a duplication (blue arrowheads) of the terminal part of the q arm. The SMC on the right side of the panel corresponds to the one described by Ventura and colleagues. (TIF) [file pgen.1008075.s005.tif]
