# Supplementary material for: Genomic inversions and GOLGA core duplicons underlie disease instability at the 15q25 locus
Source: PLoS Genet. 2019 Mar 27;15(3):e1008075. doi: 10.1371/journal.pgen.1008075 (PMC6436712; doi:10.1371/journal.pgen.1008075)
Supplement: S1 File — (PDF) [file pgen.1008075.s006.pdf]

BLAST 2 sequences results

Query ID lcl|Query_175919
Description BP1_new_chr15:82478935-82498934
Molecule type nucleic acid
Query Length 20000

Subject ID lcl|Query_175921
Description BP2_new_chr15:84404774-84506312
Molecule type nucleic acid
Subject Length 101539
Program BLASTN 2.8.1+

Graphic Summary

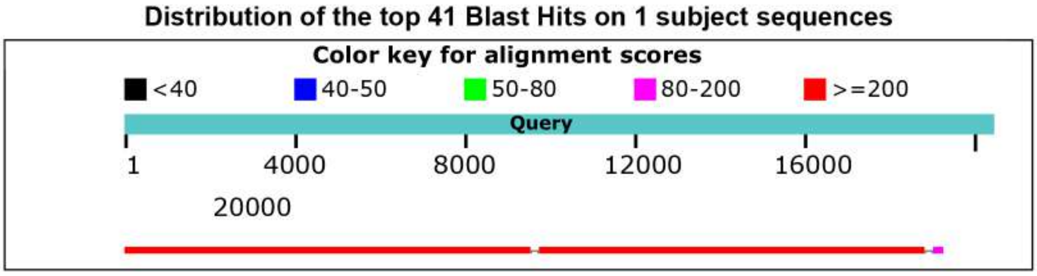

Dot Matrix View

Plot of Icl|Query_175919 vs Icl|Query_175921

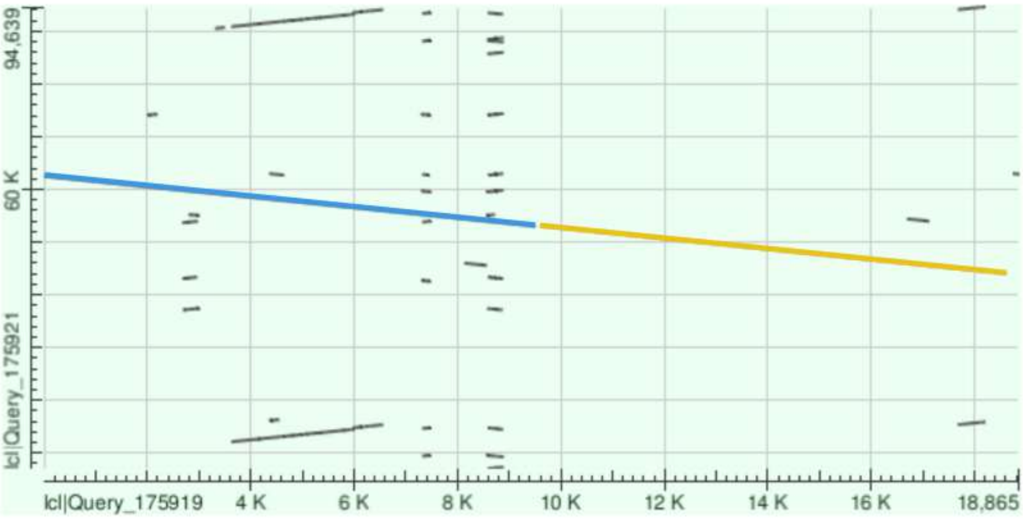

Descriptions

Sequences producing significant alignments:

Description	Max score	Total score	Query cover	E value	Ident	Accession
BP2_new_chr15:84404774-84506312 5'pad=0 3'pad=0 strand=+ repeatMasking=none	16755	48777	93%	0.0	99%	Query_175921

Alignments

1) BLAST2SeqA

BP2_new_chr15:84404774-84506312 5'pad=0 3'pad=0 strand=+ repeatMasking=none
Sequence ID: Query_175921 Length: 101539 Number of Matches: 41
Range 1: 53057 to 62500

Score	Expect	Identities	Gaps	Strand	Frame
16755 bits(9073)	0.0()	9374/9504(99%)	81/9504(0%)	Plus/Minus	

Features:

```
Query  1      CAGTTCCCAGGTTTAGGCATATGTGACTAGATAGAGTGCTAGGAGATGGATACGTGAAAA  60
              ||||||||||||||||||||||||||||||||||||||||||||||||||||||||||||
Sbjct  62500  CAGTTCCCAGGTTTAGGCATATGTGACTAGATAGAGTGCTAGGAGATGGATACGTGAAAA  62441
Query  61     TTTAAATATCATCATTTTGAACACCCATGTCACTCCAAGTGAGATTCCCTAAATATATGA  120
              ||||||||||||||||||||||||||||||||||||||||||||||||||||||||||||
Sbjct  62440  TTTAAATATCATCATTTTGAACACCCATGTCACTCCAAGTGAGATTCCCTAAATATATGA  62381
Query  121    TATACAGACAGATATATGGGTTTGAAACTCTGGAGATGAATACAAATTTAGGAGTCCCTG  180
              ||||||||||||||||||||||||||||||||||||||||||||||||||||||||||||
Sbjct  62380  TATACAGACAGATATATGGGTTTGAAACTCTGGAGATGAATACAAATTTAGGAGTCCCTG  62321
Query  181    GAACACAGGTCATGACTTAAGTAATGGGAGTCAAAGATTACTCagagaaagcacagaatg  240
              ||||||||||||||||||||||||||||||||||||||||||||||||||||||||||||
Sbjct  62320  GAACACAGGTCATGACTTAAGTAATGGGAGTCAAAGATTACTCAGAGAAAGCACAGAATG  62261
Query  241    agaagagaagaagtaggacaaggaagaagagatcggaggagaccaagaaaggGTGATAAG  300
              ||||||||||||||||||||||||||||||||||||||||||||||||||||||||||||
Sbjct  62260  AGAAGAGAAGAAGTAGGACAAGGAAGAAGAGATCGGAGGAGACCAAGAAAGGGTGATAAG  62201
Query  301    ATCAAAACAGGAGAAAAGAATCCGACAGAAGTCTCATTTGATTATCATGTCCCTTCCCAG  360
              ||||||||||||||||||||||||||||||||||||||||||||||||||||||||||||
Sbjct  62200  ATCAAAACAGGAGAAAAGAATCCGACAGAAGTCTCATTTGATTATCATGTCCCTTCCCAG  62141
Query  361    AGGACAGGGACATGTCTTTTTTGTCTTTTATACCCAATTATCACAGGTCCTGGTGCAGCA  420
              ||||||||||||||||||||||||||||||||||||||||||||||||||||||||||||
Sbjct  62140  AGGACAGGGACATGTCTTTTTTGTCTTTTATACCCAATTATCACAGGTCCTGGTGCAGCA  62081
Query  421    GACACACAGttttttttttttttAATTGTGTTGTACTATTCACAGTTTCCTGTATTCACC  480
              |||||||||||||||||||||| |||||||||||||||||||||||||||||||||||||
Sbjct  62080  GACACACAGTTTTTTTTTTTTT-AATTGTGTTGTACTATTCACAGTTTCCTGTATTCACC  62022
```

```
Query  481    AGGGGAGAAAAAAGTAAGTATAAAGAAGCACAGACACAGATGTTTTTACACTGTGTACTA  540
Sbjct  62021  AGGGGAGAAAAAAGTAAGTATAAAGAAGCACAGACACAGATGTTTTTACACTGTGTACTA  61962
Query  541    AAGGGGTCAGATTATACACAATATTTTATGCCTTACTTTTTTACTTAATATATCTTAGAA  600
Sbjct  61961  AAGGGGTCAGATTATACACAATATTTTATGCCTTACTTTTTTACTTAATATATCTTAGAA  61902
Query  601    GTTTGCACGTGCTCTTATGGAAAGACTGGCTGCATTTTTTGGTCCACAACAGAACAACAG  660
Sbjct  61901  GTTTGCACGTGCTCTTATGGAAAGACTGGCTGCATTTTTTGGTCCACAACAGAACAACAG  61842
Query  661    AATATT-----ATAAAACGGTACACTATAATTTTTATTTAACCAACTCTTTATTGGTGGA  715
Sbjct  61841  AATATTCTATTATAAAACGGTACACTATAATTTTTATTTAACCAACTCTTTATTGGTGGA  61782
Query  716    CATTAAGAATGGAGGAATGTTTCAACAAAGGAACAATCAACAGTATCAAAATACTGCAGA  775
Sbjct  61781  CATTAAGAATGGAGGAATGTTTCAACAAAGGAACAATCAACAGTATCAAAATACTGCAGA  61722
Query  776    GGGGTCAATTTGGGGACTAAGAGGGGAGCCACTGGATTTGACAACTAGGAGATAAATTTT  835
Sbjct  61721  GGGGTCAATTTGGGGACTAAGAGGGGAGCCACTGGATTTGACAACTAGGAGATAAATTTT  61662
Query  836    AGTGCAACGATGAAGGCAGAATCCAGAGTATAATGAGCTCAGTGAAAAAAGGTGAAGACA  895
Sbjct  61661  AGTGCAACGATGAAGGCAGAATCCAGAGTATAATGAGCTCAGTGAAAAAAGGTGAAGACA  61602
Query  896    TGTAGCTTATTCTCTCAAGAAACTAGGCTATGATAAACTGGCAGAGGCTCTAAGAGTGGG  955
Sbjct  61601  TGTAGCTTATTCTCTCAAGAAACTAGGCTATGATAAACTGGCAGAGGCTCTAAGAGTGGG  61542
Query  956    AGGTGAGTTGTTTTCTCCTTCATGTAAATATATTTACCTTTTAAACACTAGGCCCAATTT  1015
Sbjct  61541  AGGTGAGTTGTTTTCTCCTTCATGTAAATATATTTACCTTTTAAACACTAGGCCCAATTT  61482
Query  1016   TATATCCTATTTCATTTAACTTTATGAACATATTtatgtatgtatgcatgtatgtatgta  1075
Sbjct  61481  TATATCCTATTTCATTTAACTTTATGAACATATTTATGTATGTATGCATGTATGTATGTA  61422
Query  1076   tCTCATGTGGTGTTTTAGACACTGAAAAATAACTCATTTCTATTATAAAACTGATATCTT  1135
Sbjct  61421  TCTCATGTGATGTTTTAGACACTGAAAAATAACTCATTTCTATTATAAAACTGATATCTT  61362
Query  1136   TAGATGTTCAGAAGCAACTTCCTAAAAGGAGGTAGCAGTAATGGAGCTATGTCTATCATT  1195
Sbjct  61361  TAGATGTTCAGAAGCAACTTCCTAAAAGGAGGTAGCAGTAATGGAGCTATGTCTATCATT  61302
Query  1196   CTTTCCCATCAACCCCCTTGATGGAGATGTAAACATGTGTCCATCAAGCCTTTAATTTTT  1255
Sbjct  61301  CTTTCCCATCAACCCCCTTGATGGAGATGTAAACATGTGTCCATCAAGCCTTTAATTTTT  61242
Query  1256   ACCTCTTATCTTCATGGCTCTCCATATAAAACTTAACTCttttttttttctatttgtata  1315
Sbjct  61241  ACCTCTTATCTTCATGGCTCTCCATACAAAACTTAACTCTTTTTTTTTTCTATTTGTATA  61182
Query  1316   tgtatatttatatgtatatctatatCgagagagagagagagagaAAGAGTCTTGCTATGT  1375
Sbjct  61181  CGTATATTTATATGTATATCTATATCGAGAGAGAGAGAGAGAGAAAGAGTCTTGCTGTGT  61122
Query  1376   TGCCCAGGCTGATCTCAAACTCCTGGGCTCAAGCAATCCTCCCACCTTGGCCTCGCAAAG  1435
Sbjct  61121  TGCCCAGGCTGATCTCAAACTCCTGGGCTCAAGCAATCCTCCCACCTTGGCCTCGCAAAG  61062
Query  1436   TGCTGGGATTACAGGCGTGAACCACTGTGCCCAGCCTCAGCCTTAACTCTTAAAATATCT  1495
Sbjct  61061  TGCTGGGATTACAGGCGTGAACCACTGTGCCCAGCCTCAGCCTTAACTCTTAAAATATCT  61002
Query  1496   TCAAACCAATATTCTTCTGTTCTAATTTTTAAGAATAGATGTGTTTAAACCAACTAACTT  1555
Sbjct  61001  TCAAACCAATATTCTTCTGTTCTAATTTTTAAGAATAGATGTGTTTAAACCAACTAACTT  60942
Query  1556   ATTTTGACAAAAATTGGAGTTAAGACTCAGACTTCCTCAAATAGTTCTCCTAAAACCATT  1615
Sbjct  60941  ATTTTGACAAAAATTGGAGTTAAGACTCAGACTTCCTCAAATAGTTCTCCTAAAACCATT  60882
Query  1616   TACAGAATAATCTATCTTTTCAGTATTAAGTTAAAATACCACCTTTTCCTTATACTAAAT  1675
Sbjct  60881  TACAGAATAATCTATCTTTTCAGTATTAAGTTAAAATACCACCTTTTCCTTATACTAAAT  60822
Query  1676   TCTCGTTTGCATGACTCTGGTTCTAAACTTCCATTGCCTTTATCTGTCTGGCCCAGGGAT  1735
Sbjct  60821  TCTCGTTTGCATGACTCTGGTTCTAAACTTCCATTGCCTTTATCTGTCTGGCCCAGGGAT  60762
Query  1736   AGTCCACAATATTTTATTTACTATCTGGTTGAAAGAGTCTATACTTTATTAATTTTTATT  1795
Sbjct  60761  AGTCCACAATATTTTATTTACTATCTGGTTGAAAGAGTCTATACTTTATTACTTTTTATT  60702
Query  1796   ACTTATTCTTCTAAACAAATTTTAGAGTCGTTTTGTCAAGTGTCAAAAATAAATCTGCCA  1855
Sbjct  60701  ACTTATTCTTCTAAACAAATTTTAGAGTCGTTTTGTCAAGTGTCAAAAATAAATCTGCCA  60642
Query  1856   GAATTTGTACTGAAATTtgtgtgtgtgtgtatatatatataatacacacacactatatat  1915
Sbjct  60641  GAATTTGTACTGAAATTTGTGTGTGTGT----ATATATATAATACACACACACTATATAT  60586
Query  1916   aaaatataaaatgtatatatacaatttatatatataaatatttataaaatatgaaatata  1975
Sbjct  60585  AAAATATAAAATGTATATATACAATTTATATATATAAATATTTATAAAATATGAAATATA  60526
Query  1976   tatatata--CACACACACTTTTCTAGttttttttttttttttttttttttttttGAGAC  2033
Sbjct  60525  TATATATATACACACACACTTTTCTAGTT--------CTTTTTTTTTTTTTTTTTGAGAC  60474
Query  2034   AGGGTCTCACTCTGTCACCTAGGCTGGAGTTCAGAGGCATGATCTCGGCTCACTGCAACC  2093
Sbjct  60473  AGGGTCTCACTCTGTCACCTAGGCTGGAGTTCAGAGGCATGATCTCGGCTCACTGCAACC  60414
Query  2094   TCTGCCTCCCAGGCTCAAGTGCTCCTCCCACCTCAGCCTCAGAAGTAGTTGGAAATACAA  2153
Sbjct  60413  TCTGCCTCCCAGGCTCAAGTGCTCCTCCCACCTCAGCCTCAGAAGTAGTTGGAAATACAA  60354
```

```
Query  2154   GTGTGTGCCACAGACACCCAGCTAATTGTCATCTACCCGCCTCAGCTTCCCAAACTGTTT   2213
Sbjct  60353  GTGTGTGCCACAGACACCCAGCTAATTGTCATCTACCCGCCTCAGCTTCCCAAACTGTTT   60294
Query  2214   GGATTACAGGTATGAGCCACTGTGCCCAGCAGAAATTACATTTACAAATTAATATGAAGA   2273
Sbjct  60293  GGATTACAGGTATGAGCCACTGTGCCCAGCAGAAATTACATTTACAAATTAATATGAAGA   60234
Query  2274   CATGGTGATAACTAACATATTTATAACATGAAATCTGCTCATCCAGGAACATAGAATGCA   2333
Sbjct  60233  CATGGTGATAACTAACATATTTATAACATGAAATCTGCTCATCCAGGAACATAGAATGCA   60174
Query  2334   AATCTTTCATTCCACTCAGCAAAATTTTGTCCTGTCCTTGATAAAAGTCCTGCACATCTA   2393
Sbjct  60173  AATCTTTCATTCCACTCAGCAAAATTTTGTCCTGTCCTTGATAAAAGTCCTGCACATCTA   60114
Query  2394   AGTTTATTCCTAGGTATTTAATTTTTGCTGAAATACCTGAAAAAAATACTTCATCACTATA   2453
Sbjct  60113  AGTTTATTCCTAGGTATTTAATTTTTGCTGAAATACCTGAAAAAAATACTTCATCACTATA   60054
Query  2454   TCTTCTATGTGATTATAGCTAACATTGGGGAAGGCTATTGATTTTTATATAAAAGAACTT   2513
Sbjct  60053  TCTTCTACGTGATTATAGCTAACACTGGGGAAGGCTATTGATTTTTATATAAAAGAACTT   59994
Query  2514   TTAACCAGTAATCTTAAAAATTGTTTTTTCTCAGTTGGTTCCTTTGGATATTTTTAGGTA   2573
Sbjct  59993  TTAACCACTAATCTTAAAAATTGTTTTT-CTCAGTTGGTTCCTTTGGATATTTTTAGGTA   59935
Query  2574   AACAATCATGTCAACTGAAAATAATGATTATTTTTCTATAAAGACTATGACATCACAGGA   2633
Sbjct  59934  AACAATCATGTCAACTGAAAATAATGATAAATTTTCTATAAAGACTATGACATCACAGGA   59875
Query  2634   AAATACAGTAAATACTTTTTAAAAGAATATAAAAGGGCCAGGCACAGTGGCTCACGCCTG   2693
Sbjct  59874  AAATACAGTAAATACTTTTTAAAAGAATATAAAAGGGCCAGGCACAGTGGCTCACGCCTG   59815
Query  2694   TAATCCCAGCACTTTGGGAGGCCAAGGTGGGCAGACCATGAGGTCAGGAGATCGAGACCA   2753
Sbjct  59814  TAATCCCAGCACTTTGGGAGGCCAAGGTGGGCAGACCATGAGGTCAGGAGATCGAGACCA   59755
Query  2754   TCCTGGCTAACACGGTGGAACCCCATCTCTACTaaaaaatacaaaaaaTTAGCCGGGAAT   2813
Sbjct  59754  TCCTGGCTAACACGGTGAAACCCCATCTCTACTAAAAAATACAAAAAATTAGCCGGGAAT   59695
Query  2814   GGTGGCGGGCGCCTGTAGTCCCAGCTACTGGGGAGGCTGAGGCAGGAGAATGGTGGGAAC   2873
Sbjct  59694  GGTGGCGGGCGCCTGTAGTCCCAGCTACTGGGGAGGCTGAGGCAGGAGAATGGTGGGAAC   59635
Query  2874   CCAGGAGGTGGAGCTTGCAGTGAGCCGAGACCACGGCACTGCACTCCAGCCTGGGTGACA   2933
Sbjct  59634  CCAGGAGGTGGAGCTTGCAGTGAGCCGAGATCACGGCACTGCACTCCAGCCTGGGTGACA   59575
Query  2934   GAGCAAGACTCTGTCTCaaaaaaaaaaaaaaaaaaaaaaaaaaaaatatatatatatata   2993
Sbjct  59574  GAGCAAGACTCTGTCTCAAAAAAAAAAAAAAAAAAAAAAAAAAAAA------------GA   59527
Query  2994   tatataaaaaaCTATAGAGAATATGACCTCAACTATTAAGCATATGTGTAAGGGTTATGT   3053
Sbjct  59526  -ATAT--AAAACTATAGAGAATATGACCTCAACTATTAAGCATATGTGTAAGGGTTATGT   59470
Query  3054   ATTTTAATAGCAAAGAAAAAATATATACTGGTAGAAAATGACCATCATGTCAACAGTCAA   3113
Sbjct  59469  ATTTTAATAGCAAAGAAAAACTATATACTGGTAGAAAATGACCATCATGTCAACAGTCAA   59410
Query  3114   TAGTGGTTATATTAGATAGAGAAATTATGGGAGACTTTAAtttttttCTTTTATCTTTTC   3173
Sbjct  59409  TAGTGGTTATATTAGATAGAGAAATTATGGGAGACTTTAATTTTTTTCTTTTATCTTTTC   59350
Query  3174   TGTACTTTACCAATTTTCTCAACAATGGTTGCTTATGAGTTTTAAAATTaaaaaaaGGTT   3233
Sbjct  59349  TGTACTTTACCAATTTTCTCAACAATGGTTGCTTATGAGTTTTAAAATTAAAAAAAGGTT   59290
Query  3234   TTAAAAATTTTTCCAACATGGAAAGTTATATTTCTTTATATACTAAAAcaaaaacaaaac   3293
Sbjct  59289  TTAAAAATTTTTCCAACATGGAAAGTTATATTTCTTTATATACTAAAACAAAAACAAAAC   59230
Query  3294   tttctatttgaatacctatggcaaaaccctatctctacaaaaaatacaaaaaaTTAGCAA   3353
Sbjct  59229  TTTCTATTTGAATACCTATGGCAAAACCCTATCTCTACAAAAAATACAAAAAATTAGCAA   59170
Query  3354   GGTGGGGTAGTACACACCTGTAGTCCCAGCTACTCTAGAGGCTGAGGTGGGAGGATCACC   3413
Sbjct  59169  GGTGGGGTAGTACACACCTGTAGTCCCAGCTACTCTGGAGGCTGAGGTGGGAGGATCACC   59110
Query  3414   TGAGTCCCCAGAGAATGAGGCTCCAGTGAGCCGTGATCATAGCACTGCATTCCAGCCTGG   3473
Sbjct  59109  TGAGTCCCCAGAGAATGAGGCTCCAGTGAGCCGTGATCATAGCACTGCATTCCAGCCTGG   59050
Query  3474   GAGACAGAGAAAGACCCCATCTCaaaaaaaaaaaaaaaagaaagaaagaaaaaaagaaaTA   3533
Sbjct  59049  GAGACAGAGAAAGACCCCATCTCAAAAAAAAAAAAAA--GAAAGAAAGCAAAAAGAAATA   58992
Query  3534   TCCACAATGATCTGAAATGCCTATCTGTATGAGACTGTCCTTTGTTCACATTTTTTCAAG   3593
Sbjct  58991  TCCATAATGATCTGAAATGCCTATCTGTATGAGACTGTCCTTTGTTCACATTTTTTCAAG   58932
Query  3594   CAAATATCACACAATAAATTGAATGCAGGTACAAATGACATATCAAACATCAAAGAAATT   3653
Sbjct  58931  CAAATATCACACAATAAATTGAATGCAGGTACAAATGACATATCAAACATCAAAGAAATT   58872
Query  3654   TGCAAAAGATGTAAGACTGTACTACCTTGGGTTTAGAAATTTTCTTATCATAAAAGCATT   3713
Sbjct  58871  TGCAAAGGACGTAAGACTGTACTACCTTGGGTTTAGAAATTTTCTTATCATAAAAGCATT   58812
Query  3714   TATAACAATATTTTGTGAGCTTTTAAGGAATATTTTAAGTATTTCTGATTTAATTTCTAG   3773
Sbjct  58811  TATAACAATATTTTGTGAGCTTTTAAGGAATATTTTAAGTATTTCTGATTTAATTTCTAG   58752
Query  3774   TGATAAATACCAATAGATATAACCCACATACACAAAAGCTCCTTGGCCCTCAATATACT   3833
Sbjct  58751  TGATAAATACCAATAGATATAACCTACATACACAAAAGCTCCTTGGCCCTCAATATACT   58692
```

```
Query  3834   TTTAAGAGTGTAAAGGAATCCTGACCCCAAAACTTTGAGAACTGCTGCCTTCCCCTCTAC  3893
Sbjct  58691  TTTAAGAGTGTAAAGGAATCCTGACCCCAAAACTTTGAGAACTGCTGCCTTCCCCTCCAC  58632
Query  3894   TTTATTCCTTCCCTAGAATTTCTTCCTTGGAAGAAACATTCCTTTGCCATTCTATGTTAA  3953
Sbjct  58631  TTTATTCCTTCCCTAGAATTTCTTCCTTGGAAGAAACATTCCTTTGCCATTCTATGTTAA  58572
Query  3954   CTTACATAGTTCCATTGAGGCCAGTTTTGCTACCTCTCTCCCATCTTTCCACATCCCTCT  4013
Sbjct  58571  CTTACATAGTTCCATTGAGGCCAGTTTTGCTACCTCTCTCCCATCTTTCCACATCCCTCT  58512
Query  4014   CTTGACACAAAACCTGACCAAAGGACTCTACCGGCCCGCCCCATTTCCAGTGATTAGCTG  4073
Sbjct  58511  CTTGACACAAAACCTGACCAAAGGACTCTACCGGCCCACCCCATTTCCAGTGATTAGCTG  58452
Query  4074   TCAGGTGGGCTAAGCCAAGAAAATCTGGGTTTTCCCTGAGACTAGACCTCTCTTTCTGGG  4133
Sbjct  58451  TCAGGTGGGCTAAGCCAAGAAAATCTGGGTTTTCCCTGACACTAGACCTCTCTTTCTGGG  58392
Query  4134   AGATACGGAATCACAGGGACAAGGCTGGCCCTGTCAGAATTCATCTTGTCTAAATGGGAA  4193
Sbjct  58391  AGATACGGAATCACAGGGACAAGGCTGGCCCTGTCAGAACTCATCTTGTCTAAATGGGAA  58332
Query  4194   GAGGTTAGGCAAGTTTCTAGAATGCCAGACTGCTTTCTAGAAAGTCAAAGATAATTATAC  4253
Sbjct  58331  GAGGTTAGGCAAGTTTCTAGAATGCCAGACTGCTTTCTAGAAAGTCAAAGATAATTATAC  58272
Query  4254   TTTCTGCCACGACTGTGAAAATGCCCATTTCATTGCACGCTTTCTAACATTTATACCAAT  4313
Sbjct  58271  TTTCTGCCACGACTGTGAAAATGCCCATTTCATTGCACGCTTTCTAACATTTATACCAAT  58212
Query  4314   CTGATAAATAAAAGCTGGTACCTAGAAGaaaaaaaGGCTGGGTATGGTGGCTCATGCCTG  4373
Sbjct  58211  CTGATAAATAAAAGCTGGTACCTAGAAGAAAAAAAGGCTGGGTATGGTGGCTCATGCCTG  58152
Query  4374   TAATCCCAGTACTTTGGGAGACCAAGGTGAGTGGATCACCTGAGGTCAGGAGTTCGAGAC  4433
Sbjct  58151  TAATCCCAGTACTTTGGGAGACCAAGGTGAGTGGATCACCTGAGGTCAGGAGTTCGAGAC  58092
Query  4434   AAGCCTGGCCAACATGATGAAACCCCATCTCTAGTAAAAACACAAACATTAGCCGGGCAT  4493
Sbjct  58091  AAGCCTGGCCAACATGATGAAACCCCATCTCTAGTAAAAACACAAACATTAGCCGGGCAT  58032
Query  4494   GGTGGCAGGCCCCTGTAATCCCAACTACTCGGGAGGCTGAGGCAAGAGGATCACTTGAAC  4553
Sbjct  58031  GGTGGCAGGCCCCTGTAATCCCAACTACTCGGGAGGCTGAGGCAAGAGAATCACTTGAAC  57972
Query  4554   CTGGGAGGTGGAGGTTTTAGTGAGCCAAGATCATGCCATTGCCCTCCAGCCTGGGTGACA  4613
Sbjct  57971  CTGGGAGGTGGAGGTTTTAGTGAGCCAAGATCATGCCATTGCCCTCCAGCCTGGGTGACA  57912
Query  4614   AGCTGAGACTTTGTCTCaaaaaaaaaaaaaaaaaaaaaGTTTCCATACAATATAATTTGT  4673
Sbjct  57911  AGGTGAGACTTTGTCTCAAAAAAAAAAAAAAA------GTTTCCATACAATATAATTTGT  57858
Query  4674   TCCATCTCTAAAAACCAATTCAGCAATAACTGAAAGCCACCACTTGGAAGGTTTCAAGGA  4733
Sbjct  57857  TCCATCTCTAAAAACCAATTCAGCAATAACTGAAAGCCACCACTTGGAAGGTTTCAAGGA  57798
Query  4734   TTTAGCTCTACCTGTTGATGATGTCCAAAGCATTAGTTAAGGTAGaaaaaaaaTacacac  4793
Sbjct  57797  TTTAGCTCTACCTGTTGATGATGTCCAAAGCATTAGTTAAGGTAGAAAAAAAATA-----  57743
Query  4794   acacacacacacacacacacacacacacacacTCACCCCTATGTAGTCAGTACCAGGAAA  4853
Sbjct  57742  ---TACACACACACACACACACACACACACACTCACCCCTATGTAGTCAGTACCAGGAAA  57686
Query  4854   CACGAAAGACTAGATGGTACAGTCATCCAACACAAAGCACACAATAACTGAAGGCACTGT  4913
Sbjct  57685  CACGAAAGACTAGATGGTACAGTCATCCAACACAAAGCACACAATAACTGAAGGCACTGT  57626
Query  4914   AGAGGAGTAACTTATGACACAGATCTACAATATTGAGTGAAAATGCAGATTACaaaaaaa  4973
Sbjct  57625  AGAGGAGTAACTTATGACACAGATCTACAATATTGAGTGAAAATGCAGATTACAAAAAAA  57566
Query  4974   aaaTCTGATTTTTTAAGGGAGAGGGAACACATACAAGCAAAGGAGAAAAGAGATGAGCAG  5033
Sbjct  57565  AA-TCTGATTTTTTAAGGGAGAGGGAACACATACAAGCAAAGGAGAAAAGAGATGAGCAG  57507
Query  5034   ATGACTGAAAGATACAAAATTCTGATAGTGGTACATTCTGAGTGGTAGAATTATCAGTAT  5093
Sbjct  57506  ATGACTGAAAGATACAAAATTCTGATAGTGGTACATTCTGAGTGGTAGAATTATCAGTAT  57447
Query  5094   TATTTTCTAGTTTTGCCTAAAAATTTTCTAAATTTCTTAAGAACTTTTTGTTATCCATAT  5153
Sbjct  57446  TATTTTCTAGTTTTGCCTAAAAATTTTCTAAATTTCTTAAGAACTTTTTGTTATCCATAT  57387
Query  5154   TATCAAATATCCATCACCCCAGGAAACTTAACCTTGAGCACAAACTCTACAACAAGTTCA  5213
Sbjct  57386  TATCAAATATCCATCACCCCAGGAAACTTAACCTTGAGCACAAACTCTACAACAAGTTCA  57327
Query  5214   ATGTTTGTTCAGTTTAATATTTAAGAGACAACCTATTTTGAAAGACATCTAAAATGATGA  5273
Sbjct  57326  ATGTTTGTTCAGTTTAATATTTAAGAGACAACCTATTTTGAAAGACATCTAAAATGATGA  57267
Query  5274   CCAATATTTAAACCTATGCATTAATATTTTTCAATCATATCCTTCACATTTTGTAATTTT  5333
Sbjct  57266  CCAATATTTAAACCTATGCATTAATATTTTTCAATCATATCCTTCACATTTTGTAATTTT  57207
Query  5334   GATAAGGTTAAGCTTTAGATCCATCTTGAAAAGATAAGCTTTCTGTTTGTCTTTAAAATA  5393
Sbjct  57206  GATAAGGTTAAGCTTTAGATCCATCTTGAAAAGATAAGTTTTCTGTTTGTCTTTAAAATA  57147
Query  5394   TGACCCACAATATGCCTGTTTTTAAACAGTGAATGATGCTCTAAAATCACAATATAAATT  5453
Sbjct  57146  TGACCCACAATATGCCTGTTTTTAAACAGTGAATGATGCTCTAAAATCACAATATAAATT  57087
Query  5454   CAGGCAGTGCTCCTTACATGGAAAGTTTAAGTACTTCTAACACTGCTCTTTTTCACTTGT  5513
Sbjct  57086  CAGGCAGTGCTCCTTACATGGAAAGTTTAAGTACTTCTAACACTGCTCTTTTTCACTTGT  57027
```

```
Query  5514   TATGAAAACACAGAACAATTATCTAAGCATCTAATTATTCAGGTCCTTTGTTTCTCCTCC  5573
Sbjct  57026  TATGAAAACACAGAACAATTATCTAAGCATCTAATTATTCAGGTCCTTTGTTTCTCCTCC  56967
Query  5574   AATCTATTAGTTTTATAGTAATTTTAGGGCCTGTGAGGATGAAGCTGTCTGTGACAGCTA  5633
Sbjct  56966  AATCTATTAGTTTTATAGTAATTTTAGGGCCTGTGAGGATGAAGCTGTCTGTGACAGCTA  56907
Query  5634   CCACAAAGGTTACTATAGGTGGACAAATTTCAAACAAGTTTATCACCACTACCATCCCCA  5693
Sbjct  56906  CCACAAAGGTTACTATAGGTGGACAAATTTCAAACAAGTTTATCACCACTACCATCCCCA  56847
Query  5694   CCATAAAACTGTCTCAATCAAGGGCAACACAATTCAATATTAGCCAAGACAACCTCTTTA  5753
Sbjct  56846  CCATAAAACTGTCTCAATCAAGGGCAACACAATTCAATATTAGCCAAGACAACCTCTTTA  56787
Query  5754   CCTGTCACTGCTTAAGAAAAGGATTTTTGGTCTTATTTAGAAATAACTTTCCGTACCTAT  5813
Sbjct  56786  CCTGTCACTGCTTAAGAAAAGGATTTTTGGTCTTATTTAGAAATAACTTTCTGTACCTAT  56727
Query  5814   TTTTCTCCATAAATCCACTGAGACCAATGTGTGGCTCTATCGCAAGCACCAGCAAGCAAA  5873
Sbjct  56726  TTTTCTCCATAAATCCACTGAGACCAATGTGTGGCTCTATCTCAAGCACCAGCAAGCAAA  56667
Query  5874   ACTGCCTGCTAGAAGGTTCAGTTTTTGTATCTTTCCAAATGTAGAACACAGCTATCTTCA  5933
Sbjct  56666  ACTGCCTGCTAGAAGGTTCAGTTTTTGTATCTTTCCAAATGTAGAACACAGCTATCTTCA  56607
Query  5934   AGGATTTCATAATTTTTTGAAAATTGATGCACAAACTTCTTGAAAGTTCAGAGACACAGC  5993
Sbjct  56606  AGGATTTCATAATTTTTTGAAAATTGATGCACAAACTTCTTGAAAGTTCAGAGACACAGC  56547
Query  5994   AGCTGTAATTATTCTGAAGGCTGGTTACGGGACACATTACCTTCATACTTTGCTGTTTAA  6053
Sbjct  56546  AGCTGTAATTCTTCTGAAGGCTGGTTATGGGACACATTACCTTCATACTTTGCTGTTTAA  56487
Query  6054   GAAATGTGGGGTGGAGAATCAAGTAAACTGATAGAATTTCCATATAAAATTCTAAGTGCT  6113
Sbjct  56486  GAAATGTGGGGTGGAGAATCAAGTAAACTGATAGAATTTCCATATAAAATTCTAAGTGCT  56427
Query  6114   CTGAGAACAAAAGAAACTTAAAATacacacacacacacacacacacacacacacacacac  6173
Sbjct  56426  CTGAGAACAAAAGAAACTTAAAATACACACACACACACACACACACACACACACACACAC  56367
Query  6174   ------------GGTTTTCCCTACTAATCATTTTACAACTAAACAACCAAGTTGCTAAAC  6221
Sbjct  56366  ACACACACACACGGTTTTCCCTACTAATCATTTTACAACTAAACAACCAAGTTGCTAAAC  56307
Query  6222   CAGAGCCCACAAAAGCAGAGTCAAAGTTCTAACACTTGGTAAAAGAAAAATGCACACATA  6281
Sbjct  56306  CAGAGCCCACAAAAGCAGAGTCAAAGTTCTAACACTTGGTAAAAGAAAAATGCACACATA  56247
Query  6282   CCCCTGTGAGCTaaaaaaaaaaaTGCTTAAGTATTCAAAGACAGCAATTACAGCTACTGA  6341
Sbjct  56246  CCCCTGTGAGCTAAAAAAAAA--TGCTTAAGTATTCAAAGACAGCAATTACAGCTACTGA  56189
Query  6342   GAACATCACTGTAAGCAAACTGAGGCAGAGAAAACAAACGTGCTGATGAGGATTTGAACC  6401
Sbjct  56188  GAACATCACTGTAAGCAAACTGAGGCAGAGAAAACAAACGTGCTGATGAGGATTTGAACC  56129
Query  6402   ACCTAAGCTGCAGAAACCCACTGGATGGTTTCCTAGGTTCCGAGTTGGCATTATCTTTCA  6461
Sbjct  56128  ACCTAAGCTGCAGAAACCCACTGGATGGTTTCCTAGGTTCCGAGTTGGCATTATCTTTCA  56069
Query  6462   GAACAATCTTCTAGAAGAGATCACATAACACTGTTACAAAGGATCTGGAGAAAGGGACCC  6521
Sbjct  56068  GAACAATCTTCTAGAAGAGATCACATAACACTGTTACAAAGGATCTGGAGAAAGGGACCC  56009
Query  6522   TGGCTTCATCACTGTGGCTCTCCAGTCATGCTTTACATTTGGCAGTGACTATCTCCATTC  6581
Sbjct  56008  TGGCTTCATCACTGTGGCTCTCCAGTCATGCTTTACATTTGGCAGTGACTATCTCCATTC  55949
Query  6582   AACTCAATTCCCTAACCCTAAACTAGCTGACATTTATCAAATACTGCCCTTTACCAGGTC  6641
Sbjct  55948  AACTCAATTCCCTAACCCTAAACTAGCTGACATTTATCAAATACTGCCCTTTACCAGGTC  55889
Query  6642   TAAGTAAGTTTAACTccccccacccccACCaaaaaaaaaTCAAGATACTAAGGGATATAC  6701
Sbjct  55888  TAAGTAAGTTTAACTCCCCCCACCCCCACCAAAAAAAAATCAAGATACTAAGGGATATAC  55829
Query  6702   TATTCACAAAAGGGAAACCTGTCTCCTCTTCATATACCTGTTCCTTTCAAGGAAGGGTAT  6761
Sbjct  55828  TATTCACAAAAGGGAAACCTGTCTCCTCTTCATATACCTGTTCCTTTCAAGGAAGGGTAT  55769
Query  6762   AAAAATGGGGATGAGGGAGGATAACCACTAGGAATTTGACCCTATATTATAAATTGGTCA  6821
Sbjct  55768  AAAAATGGGGATGAGGGAGGATAACCACTAGGAATTTGACCCTATATTATAAATTGGTCA  55709
Query  6822   GATAAATGAAAATAATTCCTCTGGACTCAAAGTGATATGGCTCTGAAAATGGGAGAAACA  6881
Sbjct  55708  GATAAATGAAAATAATTCCTCTGGACTCAAAGTGATATGGCTCTGAAAACGGGAGAAACA  55649
Query  6882   TCGGGGTCCTTTGTCTCACGCCAGTTAAACGACATGGACACACAGGAGTGGTTTTAAGGA  6941
Sbjct  55648  TCGGGGTCCTTTGTCTCACGCCAGTTAAACGACATGGACACACAGGAGTGGTTTTAAGGA  55589
Query  6942   ACAGAAAGTTTAATAGGCAAGAAAGAAGAAAGGCTCCCTGCGGTACAGAAAAAGGGGGGT  7001
Sbjct  55588  GCAGAAAGTTTAATAGACAAGAAAGAAGAAAGGCTCCCTGCGGTACAGAAAAAGGGGG-T  55530
Query  7002   CTGAACAGAGAAAAAGCCCCGTGTGTGGCAGAACAGTACTCGGTTATATTGGGAGGCTGG  7061
Sbjct  55529  CTGAACAGAGAAAAAGCCCCGTGTGTGGCAGAACAGTACTCGGTTATATTGGGAGGCTGG  55470
Query  7062   AGGAGGTGGTGTCTGATTTGCACAGGGCCCAGGGCATTGGTTTGACCAGGCAGGTCATTC  7121
Sbjct  55469  AGGAGGTGGTGTCTGATTTGCACAGGGCCCAGGGCATTGGTTTGACCAGGCAGGTCATTC  55410
Query  7122   ATGTAGCCCGAGAAAAACGTGGCCCTCCCACCCTAGCCTTTTAATATGCAAATGTAGGTC  7181
Sbjct  55409  ATGTAGCCCGAGAAAAACGTGGCCCTCCCACCCTAGCCTTTTAATATGCAAATGTAGGTC  55350
```

```
Query  7182   ACCATGTTGTCCTGCACACATGGGGTCATCTGGAGGTGTCACCTTGAGGTGGTGACTAGA  7241
              ||||||||||||||||||||||||||||||||||||||||||||||||||||||||||||
Sbjct  55349  ACCATGTTGTCCTGCACACATGGGGTCATCTGGAGGTGTCACCTTGAGGTGGTGACTAGA  55290
Query  7242   AGAAGAGGGTGGGAATCTCCATGTTGAATGGACACAGTTTCTAAGCGCTGGCATTTGCAT  7301
              ||||||||||||||||||||||||||||||||||||||||||||||||||||||||||||
Sbjct  55289  AGAAGAGGGTGGGAATCTCCATGTTGAATGGACACAGTTTCTAAGCGCTGGCATTTGCAT  55230
Query  7302   ATCAAAGCTTGGCAGCCTGTAGTCCCAGCTACTCAGGAGGCTGAGGCAGGAGATTCACTT  7361
              ||||||||||||||||||||||||||||||||||||||||||||||||||||||||||||
Sbjct  55229  ATCAAAGCTTGGCAGCCTGTAGTCCCAGCTACTCAGGAGGCTGAGGCAGGAGATTCACTT  55170
Query  7362   GAACCTGGGAGGCAGAGGCTGCAGTGAGCTGAGATCACACCACTGCACTCCAGCCTGGGT  7421
              ||||||||||||||||||||||||||||||||||||||||||||||||||||||||||||
Sbjct  55169  GAACCTGGGAGGCAGAGGCTGCAGTGAGCTGAGATCACACCACTGCACTCCAGCCTGGGT  55110
Query  7422   GACAGAGCGAGATTCCGTCTCCaaaaaaaagataaaaagaaaagaaaaagaaaTGTTTCT  7481
              ||||||||||||||||||||||||||||||||||||||||||||||||||||||||||||
Sbjct  55109  GACAGAGCGAGATTCCGTCTCCAAAAAAAAGATAAAAAGAAAAGAAAAAGAAATGTTTCT  55050
Query  7482   GGAGTTGTTTCTATTAAAAGGGAAAGCCTTACTGAGGCCTCCTTACCCTCTCTATCTGCC  7541
              ||||||||||||||||||||||||||||||||||||||||||||||||||||||||||||
Sbjct  55049  GGAGTTGTTTCTATTAAAAGGGAAAGCCTTACTGAGGCCTCCTTACCCTCTCTATCTGCC  54990
Query  7542   TAGTATAATTTCTGAATAACTCCTCTATTAAAAGTACCACTGAGGTGCTTAATATGACAT  7601
              ||||||||||||||||||||||||||||||||||||||||||||||||||||||||||||
Sbjct  54989  TAGTATAATTTCTGAATAACTCCTCTATTAAAAGTACCACTGAGGTGCTTAATATGACAT  54930
Query  7602   TTCTGATATTTCCCAATGCTCCTCCACAAATTCAATTTGGAAAGGTAATCTGTTCCAGGA  7661
              ||||||||||||||||||||||||||||||||||||||||||||||||||||||||||||
Sbjct  54929  TTCTGATATTTCCCAATGCTCCTCCACAAATTCAATTTGGAAAGGTAATCTGTTCCAGGA  54870
Query  7662   GGGCAAACCAAAGAAAAAGTCCTAGGCTCCTGAGTCAAGGCTTGTTTCTTTCCTTTGTAC  7721
              ||||||||||||||||||||||||||||||||||||||||||||||||||||||||||||
Sbjct  54869  GGGCAAACCAAAGAAAAAGTCCTAGGCTCCTGAGTCAAGGCTTGTTTCTTTCCTTTGTAC  54810
Query  7722   AAGCTGACTACTTTTTAATCATAGTAAAAATGAGAAAAATGCAAGATGAAGTTAACAGCA  7781
              ||||||||||||||||||||||||||||||||||||||||||||||||||||||||||||
Sbjct  54809  AAGCTGACTACTTTTTAATCATAGTAAAAATGAGAAAAATGCAAGATGAAGTTAACAGCA  54750
Query  7782   TTGCTTTATTTCCCATGAAAAGCTGTTATAAAGCATTCTCAAAATAAACTGTTATTCAGC  7841
              ||||||||||||||||||||||||||||||||||||||||||||||||||||||||||||
Sbjct  54749  TTGCTTTATTTCCCATGAAAAGCTGTTATAAAGCATTCTCAAAATAAACTGTTATTCAGC  54690
Query  7842   CACAAATGACACCATCACTTTTTTATTCATAGGGCACAGTATCGCTGCCAAAGAGCTTTC  7901
              ||||||||||||||||||||||||||||||||||||||||||||||||||||||||||||
Sbjct  54689  CACAAATGACACCATCACTTTTTTATTCATAGGGCACAGTATCGCTGCCAAAGAGCTTTC  54630
Query  7902   CTCTATATGCTCTCTTGCAGGGCAAAAAATATTATCTATGTTATACAGAAACACAGTaaa  7961
              ||||||||||||||||||||||||||||||||||||||||||||||||||||||||||||
Sbjct  54629  CTCTATATGCTCTCTTGCAGGGCAAAAAATATTATCTATGTTATACAGAAACACAGTAAA  54570
Query  7962   aaaaGTGATTTACTTAAGGTCCTAACTACTAAATAAAAGCTAAACTACTCACTTCCTCCT  8021
              ||||||||||||||||||||||||||||||||||||||||||||||||||||||||||||
Sbjct  54569  AAAAGTGATTTACTTAAGGTCCTAACTACTAAATAAAAGCTAAACTACTCACTTCCTCCT  54510
Query  8022   AGATTCAGAGAGAGCTCCAACATTTTCTAAAATGTGGTATCTTGTTGTTGGGGTAGGCAC  8081
              ||||||||||||||||||||||||||||||||| ||||||||||||||||||||||||||
Sbjct  54509  AGATTCAGAGAGAGCTCCAACATTTTCTAAAATTTGGTATCTTGTTGTTGGGGTAGGCAC  54450
Query  8082   TTTTTGGCAATAATGAATAGACATTTAATTAGCCAATCaaaaaaaCTTATTAGGTACAGT  8141
              ||||||||||||||||||||||||||||||||||||||||||||||||||||||||||||
Sbjct  54449  TTTTTGGCAATAATGAATAGACATTTAATTAGCCAATCAAAAAAACTTATTAGGTACAGT  54390
Query  8142   AAGTTCCTCTTCAAAGGTTTAACCTGTTCAACTTCCTTGTTCTTTGTTCCTAAGAACAAT  8201
              ||||||||||||||||||||||||||||||||||||||||||||||||||||||||||||
Sbjct  54389  AAGTTCCTCTTCAAAGGTTTAACCTGTTCAACTTCCTTGTTCTTTGTTCCTAAGAACAAT  54330
Query  8202   TTCCCTGTACCTTCTCACCCCTATTTACCTGCTTAGTTATCTGCTCAGTTACCTGCCTTG  8261
              ||||||||||||||||||||||||||||||||||||||||||||||||||||||||||||
Sbjct  54329  TTCCCTGTACCTTCTCACCCCTATTTACCTGCTTAGTTATCTGCTCAGTTACCTGCCTTG  54270
Query  8262   TAAACAACTCTTCCCGTCAGTCCCAACCTGTAACTCACATTCCCTCTCCCTTCCTTATTA  8321
              ||||||||||||||| ||||||||||||||||||||||||||||||||||||||||||||
Sbjct  54269  TAAACAACTCTTCCCATCAGTCCCAACCTGTAACTCACATTCCCTCTCCCTTCCTTATTA  54210
Query  8322   GGGAGAATATTCGCGATAGCAAATCAAGTCTGCTTAGATTGTGTAGTCCGACTCCAGCCC  8381
              ||||||||||||||||||||||||||||||||||||||||||||||||||||||||||||
Sbjct  54209  GGGAGAATATTCGCGATAGCAAATCAAGTCTGCTTAGATTGTGTAGTCCGACTCCAGCCC  54150
Query  8382   ATGTGGAAATGATAGAGAGGTAGGGACTGCGTTAGGGATATAAACTCCTGCTCTATCCTG  8441
              |||||| ||||| ||||||||||||||||||||||||||||||||||||||||||||| |
Sbjct  54149  ATGTGGGAATGACAGAGAGGTAGGGACTGCGTTAGGGATATAAACTCCTGCTCTATCCCG  54090
Query  8442   CTCGGTGTGCTCTTGCATTCGTGACTAATGCAAACAGCAGTCTTTTGCAGAAGTAAGTTG  8501
              ||||||||||||||||||||||||||||||||||||||| ||||||||||||||||||||
Sbjct  54089  CTCGGTGTGCTCTTGCATTCGTGACTAATGCAAACAGCACTCTTTTGCAGAAGTAAGTTG  54030
Query  8502   TCTTGCTGAGAAAACtttttttCCTGAGTGCTGGTTCTTCCTTGCAGCACTGATCATTTG  8561
              ||||||||||||||||||||||||||||||||||||||||||||||||||||||||||||
Sbjct  54029  TCTTGCTGAGAAAACTTTTTTTCCTGAGTGCTGGTTCTTCCTTGCAGCACTGATCATTTG  53970
Query  8562   tttctttttttctttcttttttttttttttttttttttttGAGACAGAGTTTCGCTCTGTT  8621
              |||||||||||||||||||||||||||||||||      |||||||||||||||||||||
Sbjct  53969  TTTCTTTTTTTCTTTCTTTTTTTTTTTTTTTTTT------GAGACAGAGTTTCGCTCTGTT  53916
Query  8622   GCCCAGGGTGGAGCACAGTGGCTTGATCTCAGCTCACTGCAAGCTCCACCTCCCAGGTTC  8681
              ||||||||||||||||||||||||||||||||||||||||||||||||||||||||||||
Sbjct  53915  GCCCAGGGTGGAGCACAGTGGCTTGATCTCAGCTCACTGCAAGCTCCACCTCCCAGGTTC  53856
Query  8682   ACGCCATTCTCCTGCCTCAGCCTCCTGAGTAGCTGGGACTACAGGCACCCACCATTGCGC  8741
              |||||||||||||||||||||||||||||||||||||||||||||||||||||| |||||
Sbjct  53855  ACGCCATTCTCCTGCCTCAGCCTCCTGAGTAGCTGGGACTACAGGCACCCACCACTGCGC  53796
Query  8742   CCAGCTAAtttttttgtgtttttttttttttttAGTAGAGACGGCATTTCACCATGTTAG  8801
              ||||||||||||||||| |||||||||||||  |||||||||||||||||||||||||||
Sbjct  53795  CCAGCTAATTTTTTTGTATTTTTTTTTTTTT--AGTAGAGACGGCATTTCACCATGTTAG  53738
Query  8802   CCAGGATGGTCTCGATCTCCTGACTTCATGATCCGCCCACCTCGGCCTCCCAAAGTGCTG  8861
              ||||||||||||||||||||||||||||||||||||||||||||||||||||||||||||
Sbjct  53737  CCAGGATGGTCTCGATCTCCTGACTTCATGATCCGCCCACCTCGGCCTCCCAAAGTGCTG  53678
```

```
Query  8862   GGATTACAGGGGCGAGCCACTGTGCCCAGCCTGATCATTTGTTTCTAACAATCTGGGGGC  8921
Sbjct  53677  GGATTACAGGGGCGAGCCACTGCGCCCAGCCTGATCATTTGTTTCTAACAATCTGGGGGC  53618
Query  8922   TCGTCTGGAATTCCCATTCTCCTCTGAGAAAAGGGTCTCCAGTCACCAATAGTGAGGAGA  8981
Sbjct  53617  TCGTCCGGAATTCCCATTCTCCTCTGAGAAAAGGGTCTCCAGTCACCAATAGTGAGGAGA  53558
Query  8982   AGCATCCCACTGCCTCACTGAGGTGGCCTCATGGTGAGGGATCAGGACCCACCCAGTGTG  9041
Sbjct  53557  AGCATCCCACTGCCTCATTGAGGTGGCCTCATGGTGAGGGATCAGGACCCACCCAGTGTG  53498
Query  9042   ATGAATAAACCCGGACTCTCAGCAGTCTGGAAAGGAACAGACCAACAACTTAAGAGAAAA  9101
Sbjct  53497  ATGAATAAACCCGGACTCTCAGCAGTCTGGAAAGGAACAGACCAACAACTTAAGAGAAAA  53438
Query  9102   GGATCCTCACATACCATGGTGACCAGGTAACTATGTGCACAGACCAACGTAAGAAACATC  9161
Sbjct  53437  GGATCCTCACATACCATGGTGACCAGGTAACTATGTGCACAGACCAACGTAAGAAACATC  53378
Query  9162   ACAAGAGCGACAAAGTATTTTCTTGGTGGTTGGGATATCTTGGAGATTGAAAGTGTGTGT  9221
Sbjct  53377  ACAAGAGCGACAAAGTATTTTCTTGGTGGTTGGGATATCTTGGAGATTGAAAGTGTGTGT  53318
Query  9222   TGAGACTCACAATTGAGTGCAAAGCAAGTGTTCAGTCCAGATCTGCAGTTCTGTGGTCAC  9281
Sbjct  53317  TGAGACTCACAATTGAGTGCAAAGCAAGTGTTCAGTCCAGATCTGCAGTTCTGTGGTCAC  53258
Query  9282   CTTATACAGCTTAAGGTAGCCCTTCTGTAAAGGAGTCTGGGTCAGGGGTTTCTACTGAAA  9341
Sbjct  53257  CTTATACAGCTTAAGGTAGCCCTTCTGTAAAGGAGTCTGGGTCAGGGGTTTCTACTGAAA  53198
Query  9342   CAGCCATTGCTAAGAGGAAACCAACGTTCCCATGAGGGAAGCAGCCAGAGAAGGATGAAG  9401
Sbjct  53197  CAGCCATTGCTAAGAGGAAACCAACGTTCCCGTGAGGGAAGCAGCCAGAGAAGGATGAAG  53138
Query  9402   CGAAAGGAGAAAAGTGCAAGAAACCTCCAGCAGGGGGGTTGAGCCTC--ggaaaggaaag  9459
Sbjct  53137  CGAAAGGAGAAAAGTGCAAGAAACCTCCAGCAGGGGGGTTGAGCCTCTAGGAAAGGAAAG  53078
Query  9460   ggaaaggaaagggaaagggaaagg  9483
Sbjct  53077  G-AAAGGAAAGG-AAAGG-AAAGG  53057
```

2) BLAST2SeqB

Range 2: 44083 to 53089

Score	Expect	Identities	Gaps	Strand	Frame
16434 bits(8899)	0.0()	8971/9007(99%)	0/9007(0%)	Plus/Minus	

Features:

```
Query  9604   agggaagggaagggaagggaaggaaaggaaaggTGAGAAATCTCCAGTAGGAGAGGTTGA  9663
Sbjct  53089  AGGAAAGGAAAGGAAAGGAAAGGAAAGGAAAGGTGAGAAATCTCCAGTAGGAGAGGTTGA  53030
Query  9664   GCCTTATACAAACCTCTCGTAACTGGGAAGAAATTTCTAGTAGGGGAAATTGAGCCTCAC  9723
Sbjct  53029  GCCTTATACAAACCTCTCGTAACTGGGAAGAAATTTCTAGTAGGGGAAATTGAGCCTCAC  52970
Query  9724   CCCAATCCCTTTTCAAGATGGGAAATACCTCAAGTAATGCAGGGGAGAAAAAGGATAAAG  9783
Sbjct  52969  CCCAATCCCTTTTCAAGATGGGAAATACCTCAAGTAATGCAGGGGAGAAAAAGGATAAAG  52910
Query  9784   CTAGCAACAATAACATTCCTCCTGATAGTCCCCTAGGGCTTATGCTAAAATATTGAAAAG  9843
Sbjct  52909  CTAGCAACAATAACATTCCTCCTGATAGTCCCCTAGGGCTTATGCTAAAATATTGGAAAG  52850
Query  9844   AGAGTGAAAGGACTAAATACAAGAAAAAGCAGCAAATGATAAAATATTGTTGTTTCATTT  9903
Sbjct  52849  AGAGTGAAAGGACTAAATACAAGAAAAAGCAGCAAATGATAAAATATTGTTGTTTCATTT  52790
Query  9904   GGACTCAGGAATCAATCCTGAAAAGCAAGTCAGAAATTAACTCCTCTGAGAAAGATAAGG  9963
Sbjct  52789  GGACTCAGGAATCAATCCTGAAAAGCAAGTCAGAAATTAACTCCTCTGAGAAAGATAAGG  52730
Query  9964   TCCCTGTTCCTAGACAGCTCACCAACACATGGAACTTCCTCCACCACCTTCCCCCGTCCA  10023
Sbjct  52729  TCCCTGTTCCTAGACAGCTCACCAACACATGGAACTTCCTCCACCACCTTCCCCCGTCCA  52670
Query  10024  ATATCCCTAACCTCCTTCTCCCTCAAGCAGAAGCAGTTGTCCCAGACCCTTCTCCTACCC  10083
Sbjct  52669  ATACCCCTAACCTCCCTCCCCCTCAAGCAGAGGCAGTTGTCCCAGACCCTTCTCCTACCC  52610
Query  10084  ACATTGTTCCCCCTCTTTATAACCCTGCCTCTTGGGAATTGTCCCAACAGCCTGCTCACT  10143
Sbjct  52609  ACATTGTTCCCCCTCTTTATAACCCTGCCTCTTGGGAATTGTCCCAACAGCCTGCTCACT  52550
Query  10144  ATCACCCTAAGTACTCTTCCCTGAAAGGACTTCAGTGTGAGATAGAGCAATGTAAAAGGG  10203
Sbjct  52549  ATCACCCTAAGTACTCTTCCCTGAAAGGACTTCAATGTGAGATAGAGCAATGTAAAAGGG  52490
Query  10204  ATATTCAGAACTTCCCCTTCCCCTCTACCTCGGGAGAATTAGCTCCACCTCTCTTCCCCT  10263
Sbjct  52489  ATATTCAGAACTTCCCCTTCCCCTCTACCTCGGGAGAATTAGCTCCACCTCTCTTCCCCT  52430
Query  10264  GAAGAGAGGTGTCCCTACGAGGAGGAGGTATTCACTTTGTAAATGCTCCTTTAACCAGCT  10323
Sbjct  52429  GAAGAGAGGTGTCCCTACGAGGAGGAGGTATTCACTTTGTAAATGCTCCTTTAACCAGCT  52370
Query  10324  CGGAGGTCCAAAACCTAAAAACAGAGTTCAAGCCACACTATTAGACAACTCCAGTGGAAT  10383
Sbjct  52369  CGGAGGTCCAAAACCTAAAAACAGAGTTCAAGCCACACTATTAGACAACTCCAGTGGAAT  52310
Query  10384  AGCAGATCGAATTAACCAATTTCTAGGACCACAGTTATATATACTTGGGCTGAGTTAATG  10443
Sbjct  52309  AGCAGATCGAATTAACCAATTTCTAGGACCACAGTTATATATACTTGGGCTGAGTTAATG  52250
```

```
Query  10444  TCCATCCTAGGCATCCTTTTCTCAGGGGAAGAAAGAAGCATCATCTGTAGAGCTGCTATG  10503
              ||||||||||||||||||||||||||||||||||||||||||||||||||||||||||||
Sbjct  52249  TCCATCCTAGGCATCCTTTTCTCAGGGGAAGAAAGAAGCATCATCTGTAGAGCTGCTATG  52190

Query  10504  GTACCCTGGGAACATGAACACCCTCCTGGCCAAAACATTCATGCAGCGGATCAAAAATTC  10563
              ||| ||||||||||||||||||||||||||||||||||||||||||||||||||||||||
Sbjct  52189  GTAGCCTGGGAACATGAACACCCTCCTGGCCAAAACATTCATGCAGCGGATCAAAAATTC  52130

Query  10564  CCCAACCAAGACCCCTGCTGGGACAATAATAACGCAGCCCACTGAAGAGGATACGCAAGA  10623
              ||||||||||||||||||||||||||||||||||||||||||||||||||||||||||||
Sbjct  52129  CCCAACCAAGACCCCTGCTGGGACAATAATAACGCAGCCCACTGAAGAGGATACGCAAGA  52070

Query  10624  ACTTAGGGAAATGATAATAAAAGGGATTCGGGAGTCAGTACTCCGAACCCAAAATCTTAC  10683
              ||||||||||||||||||||||||||||||||||||||||||||||||||||||||||||
Sbjct  52069  ACTTAGGGAAATGATAATAAAAGGGATTCGGGAGTCAGTACTCCGAACCCAAAATCTTAC  52010

Query  10684  TCGAGCATTCGACATACAACAAAGGAAAGATGAAGGGCCTATCGAACTTTTAGACAGGTT  10743
              ||||||||||||||||||||||||||||||||||||||||||||||||||||||||||||
Sbjct  52009  TCGAGCATTCGACATACAACAAAGGAAAGATGAAGGGCCTATCGAACTTTTAGACAGGTT  51950

Query  10744  GAAAGAACAAATGAGAAAATATGCTGGCCTAGATTTAGAAGATCCTCTTAGGCAGTGAAT  10803
              ||||||||||||||||||||||||||||||||||||||||||||||||||||||||||||
Sbjct  51949  GAAAGAACAAATGAGAAAATATGCTGGCCTAGATTTAGAAGATCCTCTTAGGCAGTGAAT  51890

Query  10804  GTTAAAGCTTCATTTTGTTACTAACAGCCAGATATCACAAGGAAATTACAAAAGATAGGA  10863
              ||||||||||||||||||||||||||||||||||||||||||||||||||||||||||||
Sbjct  51889  GTTAAAGCTTCATTTTGTTACTAACAGCCAGATATCACAAGGAAATTACAAAAGATAGGA  51830

Query  10864  AATTGGAAGGACCATCCCACGAACGAGCTTCTTAGAGAAGCTCAGAAAGTGTGTGTAAGG  10923
              ||||||||||||||||||||||||||||||||||||||||||||||||||||||||||||
Sbjct  51829  AATTGGAAGGACCATCCCACGAACGAGCTTCTTAGAGAAGCTCAGAAAGTGTGTGTAAGG  51770

Query  10924  AGGGATGAGGAGAAGCaaaaagaaaaaatgaaaaTTATGTTATCCACCTTCCAACAGGGG  10983
              ||||||||||||||||||||||||||||||||||||||||||||||||||||||||||||
Sbjct  51769  AGGGATGAGGAGAAGCAAAAAGAAAAAATGAAAATTATGTTATCCACCTTCCAACAGGGG  51710

Query  10984  GCCCCAAAGGATAAAACACACCAGTATTACTCTCTGTTACCCAGAGACCCACACACTCCC  11043
              ||||||||||||||||||||||||||||||||||||||||||||||||||||||||||||
Sbjct  51709  GCCCCAAAGGATAAAACACACCAGTATTACTCTCTGTTACCCAGAGACCCACACACTCCC  51650

Query  11044  AAACAAAGCCTCCCGAGAGCCAAAACCTATAAAGATCCTAGGCCCCCACTTCCTAAGCCA  11103
              ||||||||||||||||||||||||||||||||||||||||||||||||||||||||||||
Sbjct  51649  AAACAAAGCCTCCCGAGAGCCAAAACCTATAAAGATCCTAGGCCCCCACTTCCTAAGCCA  51590

Query  11104  TATAAAGAACATAAGGAGACAAAGCCGAGAAACCCAAAAATAGAGAGAAGAGACAGAATC  11163
              |||||||||||||||||| |||||||||||||||||||||||||||||||||||||||||
Sbjct  51589  TATAAAGAACATAAGGAGGCAAAGCCGAGAAACCCAAAAATAGAGAGAAGAGACAGAATC  51530

Query  11164  AATGCTTCAATTGTGAGAAAGTAGGCCACTTCAAGAGGTATTGTCCCAAATTAAAATCAG  11223
              ||||||||||||||||||||||||||||||||||||||||||||||||||||||||||||
Sbjct  51529  AATGCTTCAATTGTGAGAAAGTAGGCCACTTCAAGAGGTATTGTCCCAAATTAAAATCAG  51470

Query  11224  AAAGAGAAGTCGTCCCACTTACGACCTTTGAGGAGGAATAGGGGGGTTAGGGGCTCTGTC  11283
              ||||||||||||||||||||||||||||||||||||||||||||||||||||||||||||
Sbjct  51469  AAAGAGAAGTCGTCCCACTTACGACCTTTGAGGAGGAATAGGGGGGTTAGGGGCTCTGTC  51410

Query  11284  TCTTTTACCTTGAATCCCACCAAGAGCCCTTGATAAATTTAGAAGTGGAACCCAAATCCG  11343
              |||||||||||||||||||||||||||||||||||||||||||||||||||||||||| |
Sbjct  51409  TCTTTTACCTTGAATCCCACCAAGAGCCCTTGATAAATTTAGAAGTGGAACCCAAATCTG  51350

Query  11344  AGCTTATGACCTTTTTAGTAGACTCAGGAGCAGCCTGCTCCTCTGTTTGTTACCTTCCCC  11403
              ||||||||||||||||||||||||||||||||||||||||||||||||||||||||||||
Sbjct  51349  AGCTTATGACCTTTTTAGTAGACTCAGGAGCAGCCTGCTCCTCTGTTTGTTACCTTCCCC  51290

Query  11404  CACAATATAACCTGGTCCTCAGAGGAGCTTGTAGTCTCAGGGGTAAAAGGAGAGGGAATC  11463
              ||||||||||||||||||||||||||||||||||||||||||||||||||||||||||||
Sbjct  51289  CACAATATAACCTGGTCCTCAGAGGAGCTTGTAGTCTCAGGGGTAAAAGGAGAGGGAATC  51230

Query  11464  AAACTAAAAATTTTAAAAGAAACAGAAATTAGATGTAAAAACTGCTCAGCTAATGTTGAA  11523
              ||||||||||||||||||||||||||||||||||||||||||||||||||||||||||||
Sbjct  51229  AAACTAAAAATTTTAAAAGAAACAGAAATTAGATGTAAAAACTGCTCAGCTAATGTTGAA  51170

Query  11524  TTTTTGTTAATTTCAGAGGCAGGAACTAATCTATTAGGAAGAAACTTAATGTTAAAATTA  11583
              ||||||||||||||||||||||||||||||||||||||||||||||||||||||||||||
Sbjct  51169  TTTTTGTTAATTTCAGAGGCAGGAACTAATCTATTAGGAAGAAACTTAATGTTAAAATTA  51110

Query  11584  GGTATAGGTTTACGTATTGGCTCAGAAGGATTCTACACTTCATTAAACCTGCTCACCACT  11643
              ||||||||||||||||||||||||||||||||||||||||||||||||||||||||||||
Sbjct  51109  GGTATAGGTTTACGTATTGGCTCAGAAGGATTCTACACTTCATTAAACCTGCTCACCACT  51050

Query  11644  GCAGAAGAAACATACATTCATCCTGATGTTTGGGCAAGGGAAGGAAATTGGGGAAAACTC  11703
              ||||||||||||||||||||||||||||||||||||||||||||||||||||||||||||
Sbjct  51049  GCAGAAGAAACATACATTCATCCTGATGTTTGGGCAAGGGAAGGAAATTGGGGAAAACTC  50990

Query  11704  CAAATTCCCCCTATACATATAAAGTTAAAAACCCCTGGAGAAATAGTAAGAAGAAAGCAA  11763
              ||||||||||||||||||||||||||||||||||||||||||||||||||||||||||||
Sbjct  50989  CAAATTCCCCCTATACATATAAAGTTAAAAACCCCTGGAGAAATAGTAAGAAGAAAGCAA  50930

Query  11764  TATCCTATTCCTTTAGAAGGCAGAATAGGCCTGAAACCTGTAATTGAAAGCCTCATCAAG  11823
              ||||||||||||||||||||||||||||||||||||||||||||||||||||||||||||
Sbjct  50929  TATCCTATTCCTTTAGAAGGCAGAATAGGCCTGAAACCTGTAATTGAAAGCCTCATCAAG  50870

Query  11824  GATGGGCTCCTTGAACCCTGTATGTCCCCTTATAACACCCCAATACTGCCTGTGAAGAAA  11883
              ||||||||||||||||||||||||||||||||||||||||||||||||||||||||||||
Sbjct  50869  GATGGGCTCCTTGAACCCTGTATGTCCCCTTATAACACCCCAATACTGCCTGTGAAGAAA  50810

Query  11884  CCAGATAGGTCATGTCGACTAGCATAAGACCTCTGGGCCATCAACCAGACAGTCTAGACT  11943
              ||||||||||||||||||||||||||||||||||||||||||||||||||||||||||||
Sbjct  50809  CCAGATAGGTCATGTCGACTAGCATAAGACCTCTGGGCCATCAACCAGACAGTCTAGACT  50750

Query  11944  ACCCATCCTGTTGTCCCTAATCCTTAAACCATTCTCAGTAAAATTCCATATGAACATCAA  12003
              ||||||||||||||||||||||||||||||||||||||||||||||||||||||||||||
Sbjct  50749  ACCCATCCTGTTGTCCCTAATCCTTAAACCATTCTCAGTAAAATTCCATATGAACATCAA  50690

Query  12004  TGGTTTACAGTAATAGGTTTAAAAGATGCCTTTTGAGCATGCTCCTTGGATGAGGACAGC  12063
              ||||||||||||||||||||||||||||||||||||||||||||||||||||||||||||
Sbjct  50689  TGGTTTACAGTAATAGGTTTAAAAGATGCCTTTTGAGCATGCTCCTTGGATGAGGACAGC  50630

Query  12064  TGAGACATTTTTGCTTTCGAATGGGAAGATCCCCATTCTGGATGACAGCAACAGTATCGA  12123
              ||||||||||||||||||||||||||||||||||||||||||||||||||||||||||||
Sbjct  50629  TGAGACATTTTTGCTTTCGAATGGGAAGATCCCCATTCTGGATGACAGCAACAGTATCGA  50570
```

```
Query  12124  TAGACAGTTCTACCCCAGGGCTTCACAGATTCCCCTAATCTCTTTGGTCAAATTCTAGAA  12183
Sbjct  50569  TAGACAGCTCTACCCCCGGGCTTCACAGATTCCCCTAATCTCTTTGGTCAAATTCTAGAA  50510
Query  12184  CAAGTGTTAGAACAAGTTTATACCCCAAAATGTATATGTCTGCTCCAGTACGTAGATGAC  12243
Sbjct  50509  CAAGTGTTAGAACAAGTTTATACCCCAAAATGTATATGTCTGCTCCAGTACGTAGATGAC  50450
Query  12244  TTATTAATATCCGGTTAGGCTATAGAAAAGGTATCTGCTTTCTCCGTCCATATCCTTAAC  12303
Sbjct  50449  TTATTAATATCCGGTTAGGCTATAGAAAAGGTATCTGCTTTCTCCATCCATATCCTTAAC  50390
Query  12304  CATTTGTAAGGAGAGGGGCTATGGGTTTCAAAGAGAAAGCTTCAATTCATAGAGCCTGAA  12363
Sbjct  50389  CATTTGTAAGGAGAGGGGCTATGGGTTTCAAAGAGAAAGCTTCAATTCATAGAGCCTGAA  50330
Query  12364  GTTAAATACCTAGGACACTTAATAAGTAACGGCAAACGAAGGATAGGGCCTGAGAGGGTT  12423
Sbjct  50329  GTTAAATACCTAGGACACTTAATAAGTAACGGCAAACGAAGGATAGGGCCTGAGAGGGTT  50270
Query  12424  GAAAGGATTGTATCCATACCTTTGCTTAAGACTAAACAAGAACTCAGAAAATTCCTAGGG  12483
Sbjct  50269  GAAGGGATTGTATCCATACCTTTGCCTAAGACTAAACAAGAACTCAGAAAATTCCTAGGG  50210
Query  12484  ATAGCTGGATATTGCCGCTTATGGATTGACTCATATGCCCTTGTCATAAAGCCTCTCTAC  12543
Sbjct  50209  ATAGCCGGATATTGCCGCTTATGGATTGACTCATATGCCCTTGTCATAAAGCCTCTCTAC  50150
Query  12544  CTAAAAATCACCCAAGAAAAGCCTGACCCTCTCCTCTGGACTTCTGAAGAACTCCACCAG  12603
Sbjct  50149  CTAAAAATCACCCAAGAAAAGCCTGACCCTCTCCTCTGGACTTCTGAAGAACTCCACCAG  50090
Query  12604  GTTGAGGAGCTAAAACATCTGCTTATAACTGCCTCTGTTTTAGCTTTGCCTTCCCTAGAA  12663
Sbjct  50089  GTTGAGGAGCTAAAACATCTGCTTATAACTGCCTCTGTTTTAGCTTTGCCTTCCCTAGAA  50030
Query  12664  AAGCCATTTCACCTTTCTGTTAACATAAATAAGGGGGTAGCTTTAGGGGTCCTTACCCAA  12723
Sbjct  50029  AAGCCATTTCACCTTTCTGTTAACATAAATAAGGGGGTAGCTTTAGGGGTCCTTACCCAA  49970
Query  12724  GAACACGGAGGTCACCAGCAACCCATGGATCTCCTATCAAAAGTTTTAGATCCAGTAACC  12783
Sbjct  49969  GAACACGGAGGTCACCAGCAACCCATGGATCTCCTATCAAAAGTTTTAGATCCAGTAACC  49910
Query  12784  TGTGGATGGCCTGAATGTTTCAATCCATTGCAGCTACCGCCTTGTTAACTAAAGAAAGCA  12843
Sbjct  49909  TGTGGATGGCCTGAATGTTTCAATCCATTGCAGCTACCGCCTTGTTAACTAAAGAAAGCA  49850
Query  12844  GAAAACTAACCTTTGGGGGAAAGTTAGTTGTAAACATGCCCCATCAGGTTAGAGCCATCT  12903
Sbjct  49849  GAAAACTAACCTTTGGGGGAAAGTTAGTTGTAAACATGCCCCATCAGGTTAGAGCCATCT  49790
Query  12904  TAAATTAAAAGGCAGGAAGGTGGCTTACTGACTTGAGAATTTTAAAGTATGAAGCTATCC  12963
Sbjct  49789  TAAATTAAAAGGCAGGAAGGTGGCTTACTGACTTGAGAATTTTAAAGTATGAAGCTATCC  49730
Query  12964  TGTTAGAAAGAGATGATTTAACACTAACCACTGATAATTCACTTAACCCAGAGGTTTCCT  13023
Sbjct  49729  TGTTAGAAAGAGATGATTTAACACTAACCACTGATAATTCACTTAACCCAGAGGTTTCCT  49670
Query  13024  GACTGGAGATCCAAATCTAAAGAGACCTGAGCATGAGTGTTTAGATTTAATGATCATACA  13083
Sbjct  49669  GACTGGAGATTCAAATCTAAAGAGACCTGAGCATGAGTGTTTAGATTTAATGATCATACA  49610
Query  13084  AAAGTTAGGCCTGATTTAAGAGAGACCCCTTACAAAACGGGGCAGGGCTTCTTTATAGAT  13143
Sbjct  49609  AAAGTTAGGCCTGATTTAAGAGAGACCCCTTACAAAACGGGGCAGGGCTTCTTTATAGAT  49550
Query  13144  GGCTCTTCCCAAGTAATTGAAGGAAAAAGGCATAATAGGTACTCAGTAGTAGATGGGGAG  13203
Sbjct  49549  GGCTCTTCCCAAGTAATTGAAGGAAAAAGGCGTAATAGGTACTCAGTAGTAGATGGGGAG  49490
Query  13204  GCACTTGAAGAAGTAGAGTCAGGAAGCCTGCCAAATAATTGGTCTGCCCAAACATGTGAA  13263
Sbjct  49489  GCACTTGAAGAAGTAGAGTCAGGAAGCCTGCCAAATAATTGGTCTGCCCAAACATGTGAA  49430
Query  13264  TGAATTGTTTGCATTAAATCAAGCCTTAAAGCACTTGCAAAACCAAGAACGGACTATTTA  13323
Sbjct  49429  TGAATTGTTTGCATTAAATCAAGCCTTAAAGCACTTGCAAAACCAAGAACGGACTATTTA  49370
Query  13324  TACTGATTCCAAGTATGCCTTTGGGGTAGCTCACACCTTTGGAAAAATTTGGACTGAACG  13383
Sbjct  49369  TACTGATTCCAAGTATGCCTTTGGGGCAGCTCACACCTTTGGAAAAATTTGGACTGAACG  49310
Query  13384  AGATCTTATTAATAGCAAAGGCCAAGACCTGGGCCACAAAGAATTAATCACCCAAGTATT  13443
Sbjct  49309  AGATCTTATTAATAGCAAAGGCCAAGACCTGGGCCACAAAGAATTAATCACCCAAGTATT  49250
Query  13444  AGATAACCTGCAGCTGCCAGAATAGCTATTGTCCATGTTCCAGGACATCAGAAAAGTCTT  13503
Sbjct  49249  AGATAACCTGCAGCTGCCAGAATAGCTATTGTCCATGTTCCAGGACATCAGAAAGGTCTT  49190
Query  13504  TCTTTTCAAAGCGGAAGGAATAACCTAGCAGATCAAATAGCCAAACACACTGCCGTTTCC  13563
Sbjct  49189  TCTTTTCAAAGCGGAAGGAATAACCTAGCAGATCAAATAGCCAAACACACTGCCGTTTCC  49130
Query  13564  TCTGAAAATGTCTGTTTTTCACTTAGCCCCTTGCCTTCCTCCCTCGACTGCAGTCCCCAT  13623
Sbjct  49129  TCTGAAAATGCCTGTTTTTCACTTAGCCCCTTGCCTTCCTCCCTCGACTGCAGTCCCCAT  49070
Query  13624  CTTTTCTCCCGCTgaaaaggaaaaaattaataaaaataggagccaaagaaaattcagaagg  13683
Sbjct  49069  CTTTTCTCCCGCTGAAAAGGAAAAAATTAATAAAAATAGGAGCCAAAGAAAATTCAGAAGG  49010
Query  13684  gaaatGGGTGTCACCAGACCAAAGAGAAATGTTATCCAAACCCCTCATGAGGGAAATTCT  13743
Sbjct  49009  GAAATGGGTGTCACCAGACCAAAGAGAAATGTTATCCAAACCCCTCATGAGGGAAATCCT  48950
Query  13744  CTCCCATCTGCATCAAGGGACTCATTAGGGACCTCAAGCTAAGTGTGATGCAGTCCTCGG  13803
Sbjct  48949  CTTTCATCTGCATCAAGGGACTCATTAGGGACCTCAAGCTAAGTGTGATGCAGTCCTCGG  48890
```

```
Query  13804  GGTCTACAGATGTATAGGAATTTATATTTTGGCAAGACAAGTTACAGATAGTTGCCTAGT  13863
              |||||||||||||||||||||||| |||||||||||||||||||||||||||||||||||
Sbjct  48889  GGTCTACAGATGTATAGGAATTTACATTTTGGCAAGACAAGTTACAGATAGTTGCCTAGT  48830

Query  13864  ATGTAAGAAGACTAATAAGCAGATCCTCAGAAAACCACCTGTTGGAGGGAGAAATCCAGG  13923
              ||||||||||||||||||||||||||||||||||||||||||||||||||||||||||||
Sbjct  48829  ATGTAAGAAGACTAATAAGCAGATCCTCAGAAAACCACCTGTTGGAGGGAGAAATCCAGG  48770

Query  13924  ATTAAGGCTGTTCCAAAGTGTCCAAATTGATTATGCCGAAATGCCCCCAATTGGTCACTT  13983
              ||||||||||||||||| ||||||||||||||||||||||||||||||||||||||||||
Sbjct  48769  ATTAAGGCTGTTCCAAATTGTCCAAATTGATTATGCCGAAATGCCCCCAATTGGTCACTT  48710

Query  13984  AAAATATTTATTAGTGATAGATCACCTTACTCATTGGGTAGAAGCTATTCCCTTTTCAAG  14043
              ||||||||||||||||||||||||||||||||||||||||||||||||||||||||||||
Sbjct  48709  AAAATATTTATTAGTGATAGATCACCTTACTCATTGGGTAGAAGCTATTCCCTTTTCAAG  48650

Query  14044  TGCAACTGCTAGTAATGTACTCAAGGCATTAGTTGAAAATATTATACCCAGGTTTGGATT  14103
              ||||||||||||||||||||||||||||||||||||||||||||||||||||||||||||
Sbjct  48649  TGCAACTGCTAGTAATGTACTCAAGGCATTAGTTGAAAATATTATACCCAGGTTTGGATT  48590

Query  14104  AATAGAAAATGCTGATTCAGACAATAGGACTCATTTCACTGCACATGTTCTTAAGAAACT  14163
              |||||||||||||||||||||||| |||||||||||||||||||||||||||||||||||
Sbjct  48589  AATAGAAAATGCTGATTCAGACAACAGGACTCATTTCACTGCACATGTTCTTAAGAAACT  48530

Query  14164  AGCCCAAGTACTAGATATAACATGGGACTACCATAACCCCTGGCACCCACCTTCATCAGG  14223
              ||||||||||||||||||||||||||||||||||||||||||||||||||||||||||||
Sbjct  48529  AGCCCAAGTACTAGATATAACATGGGACTACCATAACCCCTGGCACCCACCTTCATCAGG  48470

Query  14224  AAGAGTAGAAAGAATGAATCAGACTCTGAAAAACCACCTAACCAAATTAGTCCTAGAGAC  14283
              ||||||||||||||||||||||||||||||||||||||||||||||||||||||||||||
Sbjct  48469  AAGAGTAGAAAGAATGAATCAGACTCTGAAAAACCACCTAACCAAATTAGTCCTAGAGAC  48410

Query  14284  TCGGTTGCCATGGACTAAATGCCTCCCCATGGTCTTGTGAAGATTCCAAACTGCCCCTAG  14343
              ||||||||||||||||||||||||||||||||||||||||||||||||||||||||||||
Sbjct  48409  TCGGTTGCCATGGACTAAATGCCTCCCCATGGTCTTGTGAAGATTCCAAACTGCCCCTAG  48350

Query  14344  GAAAGATGTCGGCTCACCTCCTTATGAAATGCTGTATGAGTTGCCTTATCTACACTCCAC  14403
              ||||||||||||||||||||||||||||||||||||||||||||||||||||||||||||
Sbjct  48349  GAAAGATGTCGGCTCACCTCCTTATGAAATGCTGTATGAGTTGCCTTATCTACACTCCAC  48290

Query  14404  TGCTGACATTCCTCGTTCGAAACAAAAGATCTGTTTCTCAAGAACTATATACTTGGTCTA  14463
              ||||||||||||||||||||||||||||||||||||||||||||||||||||||||||||
Sbjct  48289  TGCTGACATTCCTCGTTCGAAACAAAAGATCTGTTTCTCAAGAACTATATACTTGGTCTA  48230

Query  14464  TCCTCCACTTTCTCTTTCCTTAGGACTAAAGGCCTCTTGGCACATACACCACCCCTTGAA  14523
              ||||||||||||||||||||||||||||||||||||||||||||||||||||||||||||
Sbjct  48229  TCCTCCACTTTCTCTTTCCTTAGGACTAAAGGCCTCTTGGCACATACACCACCCCTTGAA  48170

Query  14524  TTTCCAGTTCACCACCACCAGCCCGGACAGTGACCACATTCTCATCAAAGGTCAGAAAGA  14583
              ||||||||||||||||||||||||||||||||||||||||||||||||||||||||||||
Sbjct  48169  TTTCCAGTTCACCACCACCAGCCCGGACAGTGACCACATTCTCATCAAAGGTCAGAAAGA  48110

Query  14584  AAGGAAGCTCAAGCCCACCTGGGAGGGACATTATCTAGTGTTTCTAATGACTGAGACAGC  14643
              ||||||||||||||||||||||||||||||||||||||||||||||||||||||||||||
Sbjct  48109  AAGGAAGCTCAAGCCCACCTGGGAGGGACATTATCTAGTGTTTCTAATGACTGAGACAGC  48050

Query  14644  CGTCCACACCACTGAAAAAGAATGGACTCACCATACCTGAGTCAAAAGAGCACCACCCAC  14703
              ||||||||||||||||||||||||||||||||||||||||||||||||||||||||||||
Sbjct  48049  CGTCCACACCACTGAAAAAGAATGGACTCACCATACCTGAGTCAAAAGAGCACCACCCAC  47990

Query  14704  TCCAGAATCATGGACAGCTATTTCAGGGCCAATTCCAACCAAGTTAAAGCTAAAACGGGT  14763
              ||||||||||||||||||||||||||||||||||||||||||||||||||||||||||||
Sbjct  47989  TCCAGAATCATGGACAGCTATTTCAGGGCCAATTCCAACCAAGTTAAAGCTAAAACGGGT  47930

Query  14764  TTGATCCTCTTATGCTATATTTCTTTTCCCCTTCTATTGCTAGTCCTCTCGTTATTAATG  14823
              ||||||||||||||||||||||||||||||||||||||||||||||||||||||||||||
Sbjct  47929  TTGATCCTCTTATGCTATATTTCTTTTCCCCTTCTATTGCTAGTCCTCTCGTTATTAATG  47870

Query  14824  TAACTAGGTCGAGCTCACCCCAAACTATTACCTTTGATGCTTGCCTTGTTATATCCTGTG  14883
              ||||||||||||||||||||||||||||||||||||||||||||||||||||||||||||
Sbjct  47869  TAACTAGGTCGAGCTCACCCCAAACTATTACCTTTGATGCTTGCCTTGTTATATCCTGTG  47810

Query  14884  GAGATCTCCAAAGTCAAAAGCAACTCTCAGCCTCAGAGAAGTATCTCCGTCCCTTTCAGA  14943
              ||| ||||||||||||||||||||||||||||||||||||||||||||||||||||||||
Sbjct  47809  GAGCTCTCCAAAGTCAAAAGCAACTCTCAGCCTCAGAGAAGTATCTCCGTCCCTTTCAGA  47750

Query  14944  CAAAAGCCTCCCCCATTACGACTCTTGTTCCTTAAGAAATGTAGGGAAACAGGCCTGCCA  15003
              ||||||||||||||||||||||||||||||||||||||||||||||||||||||||||||
Sbjct  47749  CAAAAGCCTCCCCCATTACGACTCTTGTTCCTTAAGAAATGTAGGGAAACAGGCCTGCCA  47690

Query  15004  CAGCTGGAATGATATTATGTGGACAACTGAACATCAGGGCTTTGTCAACAGGCAGTTGTA  15063
              ||||||||||||||||||||||||||||||||||||||||||||||||||||||||||||
Sbjct  47689  CAGCTGGAATGATATTATGTGGACAACTGAACATCAGGGCTTTGTCAACAGGCAGTTGTA  47630

Query  15064  AGTCTCTAAAACCATGTTTGCTTTGTTAAAGGAAACATTCCCCACCCCCTGACTGCCAGT  15123
              ||||||||||||||||||||||||||||||||||||||||||||||||||||||||||||
Sbjct  47629  AGTCTCTAAAACCATGTTTGCTTTGTTAAAGGAAACATTCCCCACCCCCTGACTGCCAGT  47570

Query  15124  ATAACCAATGTAATCCAGTGCAAATTTCTATTCTTATCCCCACTTCTGCCAACCCTAAAC  15183
              ||||||||||||||||||||||||||||||||||||||||||||||||||||||||||||
Sbjct  47569  ATAACCAATGTAATCCAGTGCAAATTTCTATTCTTATCCCCACTTCTGCCAACCCTAAAC  47510

Query  15184  CTACTTTAAGTCGCTTATACGGCATAGGAGCCAAAATAGCAGGGACACATCTTATAGAAT  15243
              ||||||||||||||||||||||||||||||||||||||||||||||||||||||||||||
Sbjct  47509  CTACTTTAAGTCGCTTATACGGCATAGGAGCCAAAATAGCAGGGACACATCTTATAGAAT  47450

Query  15244  CCTTTGAAATGCATTTCATTACTTTCTCACCTCCTCCACCTCCTTCTACACTCTCTCTCA  15303
              ||||||||||||||||||||||||||||||||||||||||||||||||||||||||||||
Sbjct  47449  CCTTTGAAATGCATTTCATTACTTTCTCACCTCCTCCACCTCCTTCTACACTCTCTCTCA  47390

Query  15304  ACGAAACCGCTGTTCTTCCTTCAACCAAGGATAAAATCAAGGTAAGCCATTGTAGAAGTT  15363
              ||||||||||||||||||||||||||||||||||||||||||||||||||||||||||||
Sbjct  47389  ACGAAACCGCTGTTCTTCCTTCAACCAAGGATAAAATCAAGGTAAGCCATTGTAGAAGTT  47330

Query  15364  AAAAATTTGAAACAAACCATAGCAACTGAGACAGGGTACCAAGATGCAAATGCTTGGTTA  15423
              ||||||||||||||||||||||||||||||||||||||||||||||||||||||||||||
Sbjct  47329  AAAAATTTGAAACAAACCATAGCAACTGAGACAGGGTACCAAGATGCAAATGCTTGGTTA  47270

Query  15424  GAATGGATTAAATATTCTGTCCGCACTCTAAACAAAAGCAACTGTTACACTTGTGCGCAC  15483
              ||||||||||||||||||||||||||||||||||||||||||||||||||||||||||||
Sbjct  47269  GAATGGATTAAATATTCTGTCCGCACTCTAAACAAAAGCAACTGTTACACTTGTGCGCAC  47210
```

```
Query  15484  AGTAGGCCAGAGGCCCAGGTTGTCCCCTTTCCACTCGGATGGTCTTCCAGCCAACTGGGC  15543
              ||||||||||||||||||||||||||||||||||||||||||||||||||||||||||||
Sbjct  47209  AGTAGGCCAGAGGCCCAGGTTGTCCCCTTTCCACTCGGATGGTCTTCCAGCCAACTGGGC  47150

Query  15544  ATGAGCTGTATGGTAGCTCTTCTCCAAGACCCCACAGCCTGGGGTAATGAATCTTGCCAA  15603
              ||||||||||||||||||||||||||||||||||||||||||||||||||||||||||||
Sbjct  47149  ATGAGCTGTATGGTAGCTCTTCTCCAAGACCCCACAGCCTGGGGTAATGAATCTTGCCAA  47090

Query  15604  GCTCTCTCTCTGCTATTCCCTAAAGTCCAACACCCTGCAGGTCAGTCCCTGAGGGCCATC  15663
              ||||||||||||||||||||||||||||||||||||||||||||||||||||||||||||
Sbjct  47089  GCTCTCTCTCTGCTATTCCCTAAAGTCCAACACCCTGCAGGTCAGTCCCTGAGGGCCATC  47030

Query  15664  CAGCCTCCATCTATTGACACCAATTTTTACCTCGGGTCTCTCACAACAAGGGGAAAACTT  15723
              ||||||||||||||||||||||||||||||||||||||||||||||||||||||||||||
Sbjct  47029  CAGCCTCCATCTATTGACACCAATTTTTACCTCGGGTCTCTCACAACAAGGGGAAAACTT  46970

Query  15724  GGCATTTCATGAAGACCTAAAGGGATGCGGTGAACTTAAACTCTCCCAAGAGCTTACCAG  15783
              ||||||||||||||||||||||||||||||||||||||||||||||||||||||||||||
Sbjct  46969  GGCATTTCATGAAGACCTAAAGGGATGCGGTGAACTTAAACTCTCCCAAGAGCTTACCAG  46910

Query  15784  TCAGTCTGCCCTTGTTCATCCTCGAGCATACGTATGGTGGTATTGTGGTGGACCCTTACT  15843
              ||||||||||||||||||||||||||||||||||||||||||||||||||||||||||||
Sbjct  46909  TCAGTCTGCCCTTGTTCATCCTCGAGCATACGTATGGTGGTATTGTGGTGGACCCTTACT  46850

Query  15844  GGACACTCTGCCAAGCAACTGGAGTGGTACTTGTGCTCTAGTCCAACTGGCCATCCCTTT  15903
              ||||||||||||||| ||||||||||||||||||||||||||||||||||||||||||||
Sbjct  46849  GGACACTCTGCCAAGTAACTGGAGTGGTACTTGTGCTCTAGTCCAACTGGCCATCCCTTT  46790

Query  15904  CACCCTAGCATTCCATTAACATAATAGAAGAGAAAATCAGAAGAGAAGAAGTGACCTTCA  15963
              ||||||||||||||||||||||||||||||||||||||||||||||||||||||||||||
Sbjct  46789  CACCCTAGCATTCCATTAACATAATAGAAGAGAAAATCAGAAGAGAAGAAGTGACCTTCA  46730

Query  15964  TGGGTCCTTTGACTCCCACGTTTATAAAGATGCTACTGGAGTTCCACGAGGGGTACCAGA  16023
              ||||||||||||||||||||||||||||||||||||||||||||||||||||||||||||
Sbjct  46729  TGGGTCCTTTGACTCCCACGTTTATAAAGATGCTACTGGAGTTCCACGAGGGGTACCAGA  46670

Query  16024  TAAATTTAAGGCCCGAAATCAAACAGCTTCAGGATTTGAATCTGTGCTGTTTTGGTGGTC  16083
              ||||||||||||||||||||||||||||||||||||||||||||||||||||||||||||
Sbjct  46669  TAAATTTAAGGCCCGAAATCAAACAGCTTCAGGATTTGAATCTGTGCTGTTTTGGTGGTC  46610

Query  16084  AACTGTAAATAAAAATGTAGATCGGATAAACTACATTTATTACAACCAACAAAGGTTTGT  16143
              ||||||||||||||||||||||||||||||||||||||||||||||||||||||||||||
Sbjct  46609  AACTGTAAATAAAAATGTAGATCGGATAAACTACATTTATTACAACCAACAAAGGTTTGT  46550

Query  16144  TAACTACACAAGACATGCCATTAAGGGAACAGCCTCCCAATTAGGTCCCATTAACTAAAT  16203
              ||||||||||||||||||||||||||||||||||||||||||||||||||||||||||||
Sbjct  46549  TAACTACACAAGACATGCCATTAAGGGAACAGCCTCCCAATTAGGTCCCATTAACTAAAT  46490

Query  16204  AGTCTGGGAAAACAGGATAGCCCTAGATACGATGCTAGCAGAAAAAGGTGGTGTCTGTGT  16263
              ||||||||||||||||||||||||||||||||||||||||||||||||||||||||||||
Sbjct  46489  AGTCTGGGAAAACAGGATAGCCCTAGATACGATGCTAGCAGAAAAAGGTGGTGTCTGTGT  46430

Query  16264  CATGATTGGAGTCCAATGATGTACTTTTATTCCTAATAACACAGCCCCTGACGGAACAGT  16323
              ||||||||||||||||||||||||||||||||||||||||||||||||||||||||||||
Sbjct  46429  CATGATTGGAGTCCAATGATGTACTTTTATTCCTAATAACACAGCCCCTGACGGAACAGT  46370

Query  16324  AACAAAAGCTTTGCAGGACCTAACCTCCTTATCCAATGAGTTAGCAAGCAATTCTGGAAT  16383
              ||||||||||||||||||||||||||||||||||||||||||||||||||||||||||||
Sbjct  46369  AACAAAAGCTTTGCAGGACCTAACCTCCTTATCCAATGAGTTAGCAAGCAATTCTGGAAT  46310

Query  16384  AAATGATCCCTTTACAAGTTTAATGGAGAAATGGTCTGGAAAATGGAAAGGCTTAATGTC  16443
              ||||||||||||||||||||||||||||||||||||||||||||||||||||||||||||
Sbjct  46309  AAATGATCCCTTTACAAGTTTAATGGAGAAATGGTCTGGAAAATGGAAAGGCTTAATGTC  46250

Query  16444  CTCAATATTTACTTCTCTTGCAATCGTTATAGGTGTGCTTATTCTTGTTGGATGCTGTAT  16503
              ||||||||||||||||||||||||||||||||||||||||||||||||||||||||||||
Sbjct  46249  CTCAATATTTACTTCTCTTGCAATCGTTATAGGTGTGCTTATTCTTGTTGGATGCTGTAT  46190

Query  16504  CATACCATACATTTGTGGACTACTGCAAAGACTCATAGACACAGAACTTACCAAAACCTC  16563
              ||||||||||||||||||||||||||||||||||||||||||||||||||||||||||||
Sbjct  46189  CATACCATACATTTGTGGACTACTGCAAAGACTCATAGACACAGAACTTACCAAAACCTC  46130

Query  16564  TCTTAGCTCTCCTCCACCCTATTCAGATAAGCTTTTCCTTCTAGAAAACCAAGCAGAACA  16623
              ||||||||||||||||||||||||||||||||||||||||||||||||||||||||||||
Sbjct  46129  TCTTAGCTCTCCTCCACCCTATTCAGATAAGCTTTTCCTTCTAGAAAACCAAGCAGAACA  46070

Query  16624  GCAAAGCAAAGACATGCTAAAAAAGTTTGAAGAGGAAGAATTACAAAAATTAAGAGGGGG  16683
              ||||||||||||||||||||||||||||||||||||||||||||||||||||||||||||
Sbjct  46069  GCAAAGCAAAGACATGCTAAAAAAGTTTGAAGAGGAAGAATTACAAAAATTAAGAGGGGG  46010

Query  16684  GAATTGTTAGGTACAGTAAGTTCCTCTTCAAAGGTTTAACTTGTTCAACTTCCTTGTTCT  16743
              ||||||||||||||||||||||||||||||||||||||||||||||||||||||||||||
Sbjct  46009  GAATTGTTAGGTACAGTAAGTTCCTCTTCAAAGGTTTAACTTGTTCAACTTCCTTGTTCT  45950

Query  16744  CTGTTCCTAAGAACAATTTCCCTGTACCTTCTCGACCCTACTTACCAGCTTAGTTACCTG  16803
              ||||||||||||||||||||||||||||||||||||||||||||||||||||||||||||
Sbjct  45949  CTGTTCCTAAGAACAATTTCCCTGTACCTTCTCGACCCTACTTACCAGCTTAGTTACCTG  45890

Query  16804  CTTAGTAACCTGCCTTGTAAACAACTCTTCCTACCAGCCCCAATCTGTAACTCACATTCC  16863
              ||||||||||||||||||||||||||||||||||||||||||||||||||||||||||||
Sbjct  45889  CTTAGTAACCTGCCTTGTAAACAACTCTTCCTACCAGCCCCAATCTGTAACTCACATTCC  45830

Query  16864  CCCTCCCTTTCTTATTAGAGAAAATATTCACAATATCCAGCTGAGTCAGCTAAGATTGTG  16923
              ||||||||||||||||||||||||||||||||||||||||||||||||||||||||||||
Sbjct  45829  CCCTCCCTTTCTTATTAGAGAAAATATTCACAATATCCAGCTGAGTCAGCTAAGATTGTG  45770

Query  16924  CAGTCCTACCCCAGCCCATGTTGGAATGACACAGAGGTAGGGAGTGCATTAGGGATAAGA  16983
              ||||||||||||||||||||||||||||||||||||||||||||||||||||||||||||
Sbjct  45769  CAGTCCTACCCCAGCCCATGTTGGAATGACACAGAGGTAGGGAGTGCATTAGGGATAAGA  45710

Query  16984  ACCCCTGCTCCACCCCGTTTGGTGTGCTCTTGCAATCATGACTAATGCAAGCAGCATACT  17043
              ||||||||||||||||||||||||||||||||||||||||||||||||||||||||||||
Sbjct  45709  ACCCCTGCTCCACCCCGTTTGGTGTGCTCTTGCAATCATGACTAATGCAAGCAGCATACT  45650

Query  17044  TGCAGAAGCAAATTGTCTTGCTGAGAAAACTTTTTTGCCTGAGTGCTGCTTCTTCCTCAC  17103
              ||||||||||||||||||||||||||||||||||||||||||||||||||||||||||||
Sbjct  45649  TGCAGAAGCAAATTGTCTTGCTGAGAAAACTTTTTTGCCTGAGTGCTGCTTCTTCCTCAC  45590

Query  17104  AGCACCGATCATTTGTTTCTAACAATCTCGCTAAAAGCAGCCTAGAAAGCAGCCACTTAT  17163
              |||||| |||||||||||||||||||||||||||||||||||||||||||||||||||||
Sbjct  45589  AGCACCAATCATTTGTTTCTAACAATCTCGCTAAAAGCAGCCTAGAAAGCAGCCACTTAT  45530
```

```
Query  17164  GCAGAAAGAGTAATAATTTATGCTCTACAAGTCATATAAAAAATGAAGTTTCATTTGTTT  17223
              ||||||||||||||||||||||||||||||||||||||||||||||||||||||||||||
Sbjct  45529  GCAGAAAGAGTAATAATTTATGCTCTACAAGTCATATAAAAAATGAAGTTTCATTTGTTT  45470

Query  17224  ACCGGCTAATTTACTTCCTGGGAGACATTTTTCATTCTAAAACAGTGATTCCCTACCAAG  17283
              ||||||||||||||||||||||||||||||||||||||||||||||||||||||||||||
Sbjct  45469  ACCGGCTAATTTACTTCCTGGGAGACATTTTTCATTCTAAAACAGTGATTCCCTACCAAG  45410

Query  17284  AGTTCATAGATGCCAAGAAGTCCATAAAAGGCGTAATGGAATTGCCAAATTATGTTAAAT  17343
              ||||||||||||||||||||||||||||||||||||||||||||||||||||||||||||
Sbjct  45409  AGTTCATAGATGCCAAGAAGTCCATAAAAGGCGTAATGGAATTGCCAAATTATGTTAAAT  45350

Query  17344  ACTTCAAAAGGACTCAAAGCCATATACTAGTTCCCAATAGGCCTGCACAAGTTATTAGAA  17403
              ||||||||||||||||||||||||||||||||||||||||||||||||||||||||||||
Sbjct  45349  ACTTCAAAAGGACTCAAAGCCATATACTAGTTCCCAATAGGCCTGCACAAGTTATTAGAA  45290

Query  17404  CAAGCTGCTTTGCATTCTTGTGTGATCAGAACCAGTAACTAGATGGCAATCAGGTCTGTT  17463
              ||||||||||||||||||||||||||||||||||||||||||||||||||||||||||||
Sbjct  45289  CAAGCTGCTTTGCATTCTTGTGTGATCAGAACCAGTAACTAGATGGCAATCAGGTCTGTT  45230

Query  17464  ACTGAAGATGGAAAAACTATACTTAAGTTTGTATAACAATCTTTCATAACATGGCTTCAC  17523
              ||||||||||||||||||||||||||||||||||||||||||||||||||||||||||||
Sbjct  45229  ACTGAAGATGGAAAAACTATACTTAAGTTTGTATAACAATCTTTCATAACATGGCTTCAC  45170

Query  17524  AGAAAAGAAGTATAAAAAGGATTCCTTGGTTGAAAAAGAGTGCTCTTTTCCCTTCATTAT  17583
              ||||||||||||||||||||||||||||||||||||||||||||||||||||||||||||
Sbjct  45169  AGAAAAGAAGTATAAAAAGGATTCCTTGGTTGAAAAAGAGTGCTCTTTTCCCTTCATTAT  45110

Query  17584  TTAAGATTAGGACAAATTTATAAAACAGGaaaaaaaTAGCACAAATCCCTTGGCAAACAG  17643
              |||||||||||||||||||||||||||||       ||||||||||||||||||||||||
Sbjct  45109  TTAAGATTAGGACAAATTTATAAAACAGGAAAAAAATAGCACAAATCCCTTGGCAAACAG  45050

Query  17644  AGTAAAACATCTACTCTGTTTTGCtttttttCACTTCTTACACTCTCTTTCATAGGAAGT  17703
              ||||||||||||||||||||||||       |||||||||||||||||||||||||||||
Sbjct  45049  AGTAAAACATCTACTCTGTTTTGCTTTTTTTCACTTCTTACACTCTCTTTCATAGGAAGT  44990

Query  17704  CAATTTACAGACTTCCATCAAGCCCTTAGAGACCTTTTTGTACTATCCATGACAAGCTCT  17763
              ||||||||||||||||||||||||||||||||||||||||||||||||||||||||||||
Sbjct  44989  CAATTTACAGACTTCCATCAAGCCCTTAGAGACCTTTTTGTACTATCCATGACAAGCTCT  44930

Query  17764  TGATGTTATCTCTGCACTTTTGACAAATTCTTAGCAGTTAACTTACAAGGCAGTTAAGAT  17823
              ||||||||||||||||||||||||||||||||||||||||||||||||||||||||||||
Sbjct  44929  TGATGTTATCTCTGCACTTTTGACAAATTCTTAGCAGTTAACTTACAAGGCAGTTAAGAT  44870

Query  17824  TTTTGTTCAAGCACAATATAGCTAGAATAGGCTCATACATTCAATAAAACAAATATTTAC  17883
              ||||||||||||||||||||||||||||||||||||||||||||||||||||||||||||
Sbjct  44869  TTTTGTTCAAGCACAATATAGCTAGAATAGGCTCATACATTCAATAAAACAAATATTTAC  44810

Query  17884  CAAGCATTTATTGAGTGGAAGATAAAAAGCACAAAGCATAATTATAAAATATTTTCCCCT  17943
              ||||||||||||||||||||||||||||||||||||||||||||||||||||||||||||
Sbjct  44809  CAAGCATTTATTGAGTGGAAGATAAAAAGCACAAAGCATAATTATAAAATATTTTCCCCT  44750

Query  17944  GCCACCATaaaaaaaTTAAACAGGCTTACAGAATACAGTGTAAGAAAACATGACCAAAGC  18003
              ||||||||       |||||||||||||||||||||||||||||||||||||||||||||
Sbjct  44749  GCCACCATAAAAAAATTAAACAGGCTTACAGAATACAGTGTAAGAAAACATGACCAAAGC  44690

Query  18004  AAAAATAGTAAGGACTAAAGAAGGGAGGAAGGGGAAATATCAACATGGACTGAATATGAC  18063
              ||||||||||||||||||||||||||||||||||||||||||||||||||||||||||||
Sbjct  44689  AAAAATAGTAAGGACTAAAGAAGGGAGGAAGGGGAAATATCAACATGGACTGAATATGAC  44630

Query  18064  CCAAAAGAGCCTTGATGGATGGTCAGACATGTAAAGGCAAATTGGTTAGGGTTAAGGGGT  18123
              ||||||||||||||||||||||||||||||||||||||||||||||||||||||||||||
Sbjct  44629  CCAAAAGAGCCTTGATGGATGGTCAGACATGTAAAGGCAAATTGGTTAGGGTTAAGGGGT  44570

Query  18124  GGAGGTCAGGGCACGTTCTATAGGGAAACGGCAGCTGATACAGAAGCCTGAAAGGAAAAG  18183
              ||||||||||||||||||||||||||||||||||||||||||||||||||||||||||||
Sbjct  44569  GGAGGTCAGGGCACGTTCTATAGGGAAACGGCAGCTGATACAGAAGCCTGAAAGGAAAAG  44510

Query  18184  CGGCAGAGCACCTGGACAGGCCTCTTCAGGAACGAGCACGCACGTGCGTGAAAAACAAC  18243
              |||||||||||||||||||| ||||||||||||||||||||||||||||||||||||||
Sbjct  44509  CGGCAGAGCACCTGGACAGGACTCTTCAGGAACGAGCACGCACGTGCGTGAAAAACAAC  44450

Query  18244  TTAGTGAGGTACCGTTCACCCAAACATTAGAGAAACCGCGTAAAAATGCTTCTTGGTAAG  18303
              ||||||||||||||||||||||||||||||||||||||||||||||||||||||||||||
Sbjct  44449  TTAGTGAGGTACCGTTCACCCAAACATTAGAGAAACCGCGTAAAAATGCTTCTTGGTAAG  44390

Query  18304  CATGAAGAAGGCAGGGCTCGCCCTGTAGAAGAACTCAATAAACATTTGAACTGTCTAAAG  18363
              ||||||||||||||||||||||||||||||||||||||||||||||||||||||||||||
Sbjct  44389  CATGAAGAAGGCAGGGCTCGCCCTGTAGAAGAACTCAATAAACATTTGAACTGTCTAAAG  44330

Query  18364  AGTAAAAGTTAATGAATAGGCCAAACTCACTCCTTTCTTTGTTTTAAGAGCTACAACTTT  18423
              ||||||||||||||||||||||||||||||||||||||||||||||||||||||||||||
Sbjct  44329  AGTAAAAGTTAATGAATAGGCCAAACTCACTCCTTTCTTTGTTTTAAGAGCTACAACTTT  44270

Query  18424  AGAGAATAACAAATCACAAACCCAGTAGACAGGTCCTGGCATTTCAAATCCAACCCCATT  18483
              ||||||||||||||||||||||||||||||||||||||||||||||||||||||||||||
Sbjct  44269  AGAGAATAACAAATCACAAACCCAGTAGACAGGTCCTGGCATTTCAAATCCAACCCCATT  44210

Query  18484  TTTCCCTTAATCTTTCCCCTCTGAGCAAATGGTATCGACATGAACAAGCCATGTTGATTT  18543
              ||||||||||||||||||||||||||||||||||||||||||||||||||||||||||||
Sbjct  44209  TTTCCCTTAATCTTTCCCCTCTGAGCAAATGGTATCGACATGAACAAGCCATGTTGATTT  44150

Query  18544  GATCAAGACACTCATCCATGGTTAAAAGAGTCTTTACCTTCAAGAGATACAAACAGAAAT  18603
              |||||||||||||||||||||||||||||||||||||| |||||||||||||||||||||
Sbjct  44149  GATCAAGACACTCATCCATGGTTAAAAGAGTCTTTACTTTCAAGAGATACAAACAGAAAT  44090

Query  18604  ATTGACA  18610
              ||| |||
Sbjct  44089  ATTTACA  44083
```
